# Long-term exposure to ambient fine particulate components and leukocyte epigenome-wide DNA Methylation in older men: the Normative Aging Study

**DOI:** 10.1186/s12940-023-01007-5

**Published:** 2023-08-07

**Authors:** Cuicui Wang, Heresh Amini, Zongli Xu, Adjani A. Peralta, Mahdieh Danesh Yazdi, Xinye Qiu, Yaguang Wei, Allan Just, Jonathan Heiss, Lifang Hou, Yinan Zheng, Brent A. Coull, Anna Kosheleva, Andrea A. Baccarelli, Joel D. Schwartz

**Affiliations:** 1 Department of Environmental Health, Harvard T.H. Chan School of Public Health, Boston, MA 02115 USA; 2 Department of Public Health, Faculty of Health and Medical Sciences, Section of Environmental Health, University of Copenhagen, Copenhagen, Denmark; 3 Biostatistics & Computational Biology Branch, National Institute of Environmental Health Sciences, NIH, Research Triangle Park, Durham, NC USA; 4 Program in Public Health, Department of Family, Population, and Preventive Medicine, Renaissance School of Medicine at Stony Brook University, Stony Brook, NY USA; 5 Department of Environmental Medicine and Public Health, Icahn School of Medicine at Mount Sinai, New York, NY 10029 USA; 6 Department of Preventive Medicine, Feinberg School of Medicine, Northwestern University, Chicago, IL 60611 USA; 7 Department of Biostatistics, Harvard T. H. Chan School of Public Health, Boston, MA 02115 USA; 8 Department of Environmental Health Sciences, Columbia Mailman School of Public Health, New York, NY 10032 USA; 9 Department of Epidemiology, Harvard T.H. Chan School of Public Health, Boston, MA 02115 USA

**Keywords:** PM_2.5_ components, Sources, DNA methylation, Epigenome-wide association study, Pathway analyses

## Abstract

**Background:**

Epigenome-wide association studies of ambient fine particulate matter (PM_2.5_) have been reported. However, few have examined PM_2.5_ components (PMCs) and sources or included repeated measures. The lack of high-resolution exposure measurements is the key limitation. We hypothesized that significant changes in DNA methylation might vary by PMCs and the sources.

**Methods:**

We predicted the annual average of 14 PMCs using novel high-resolution exposure models across the contiguous U.S., between 2000–2018. The resolution was 50 m × 50 m in the Greater Boston Area. We also identified PM_2.5_ sources using positive matrix factorization. We repeatedly collected blood samples and measured leukocyte DNAm with the Illumina HumanMethylation450K BeadChip in the Normative Aging Study. We then used median regression with subject-specific intercepts to estimate the associations between long-term (one-year) exposure to PMCs / PM_2.5_ sources and DNA methylation at individual cytosine-phosphate-guanine CpG sites. Significant probes were identified by the number of independent degrees of freedom approach, using the number of principal components explaining > 95% of the variation of the DNA methylation data. We also performed regional and pathway analyses to identify significant regions and pathways.

**Results:**

We included 669 men with 1,178 visits between 2000–2013. The subjects had a mean age of 75 years. The identified probes, regions, and pathways varied by PMCs and their sources. For example, iron was associated with 6 probes and 6 regions, whereas nitrate was associated with 15 probes and 3 regions. The identified pathways from biomass burning, coal burning, and heavy fuel oil combustion sources were associated with cancer, inflammation, and cardiovascular diseases, whereas there were no pathways associated with all traffic.

**Conclusions:**

Our findings showed that the effects of PM_2.5_ on DNAm varied by its PMCs and sources.

**Supplementary Information:**

The online version contains supplementary material available at 10.1186/s12940-023-01007-5.

## Introduction

Ambient fine particulate matter (an aerodynamic diameter ≤ 2.5 µm; PM_2.5_) is a complex mixture of many components (PM_2.5_ components; PMCs) that differ in their physicochemical, and toxicological properties [1]. Studies have not only found that exposure to ambient PM_2.5_ was linked with death [2] and multi-systemic diseases [3–7] but have observed that PM_2.5_-related effects vary by its PMCs [8–10]. In addition, there are multiple sources of ambient PM_2.5_, including direct emissions (e.g., biomass burning, and inefficient fuel combustion) [11, 12] and secondary particles (chemical reactions of gas-phase pollutant precursors, e.g., nitrogen oxides). Studies also have suggested that PM_2.5_-related adverse health effects vary by different sources [13–15]. However, there are inconsistent results on which PMCs or PM_2.5_ sources are responsible for the adverse health effects [16–19]. Further, the underlying molecular changes caused by PMCs and the sources have not been adequately investigated.

DNA methylation (DNAm), a chemical modification of DNA with a methyl group addition predominantly at a cytosine-phosphate-guanine (CpG) site [20], has been associated with poor health outcomes, such as cardiovascular diseases (CVDs), cancer, aging, oxidative stress, and inflammation [20–24]. Meanwhile, DNAm has been linked with PM_2.5_ across different time windows [25–28]. For instance, our group have conducted an epigenome-wide association study (EWAS) of PM_2.5_ across a 28-day time window in the Normative Aging Study and found 2,717 statistically significant CpGs [29]. Since PM_2.5_ is a combination of multiple PMCs with different characteristics, these PMCs may be associated with DNAm at different sites. To date, only six studies have assessed the associations between PMCs and DNAm across different time windows [29–34], among which only one examined the long-term effects of PMCs on DNAm in an epigenome-wide scope [34] and two were conducted by our group previously [29, 34]. However, five [29–31, 33, 34] of the six studies obtained the PMCs’ concentrations from limited monitoring sites that do not reflect the spatial variation in the concentrations.

One vital limitation in studies using PMCs from fixed monitors is the lack of high-resolution exposure measurements. It leads to a low spatial resolution of PMCs in these studies despite evidence that some components (e.g., black carbon) can vary substantially over distances as small as 100 ~ 200 m [35, 36]**.** Emerging exposure prediction models which utilize methods such as chemical transport models [37, 38] and land use regression models [39, 40] provide higher exposure resolution. Nevertheless, these models have relatively moderate prediction accuracies and/or spatial resolutions. Our group recently developed novel prediction models with 50 m resolution of PMCs across the contiguous U.S, using machine learning and a mixture of land use, remote sensing, and other inputs [41]. These new models not only diminish the measurement errors for each PMC (out of sample R^2^ ~ 0.9) but minimize the extent of measurement errors across PMCs. We have applied the predicted PMCs in several epidemiological studies [42, 43]. To date, however, no EWAS of PMCs from high-resolution models nor EWAS of PM_2.5_ sources has been previously performed.

This study, therefore, sought to investigate the associations between long-term exposure to PMCs/sources and DNAm by conducting EWAS analyses, using whole-blood samples and exposure data from high-resolution models. We hypothesize that the changes in DNAm varied by PMCs and sources.

## Materials and methods

### Study population

The participants in this study included 669 elderly men in the Greater Boston Area who are part of the Normative Aging Study, a closed and ongoing cohort established by the U.S. Veterans Administration [44]. For the initial cohort, the participants were aged 21–82 years and were free of any known chronic diseases. They have physical examinations, including blood collection, and questionnaires in a clinical center every 3–5 years. In this study, we included subjects who had visits with their DNA samples collected in 2000 and later. To dimmish study heterogeneity that may be introduced by diverse genetic ancestry, we dropped non-white participants (~ 3%) [45]. The Harvard T.H. Chan School of Public Health and the Institutional Review Boards of the Department of Veterans Affairs approved the study proposal. All study participants provided their written informed consent before enrollment and at sample collection.

### PM_2.5_ and its 14 components measures

We predicted annual average ambient PMCs based on a combination of machine learning algorithms in a geographically weighted regression. The resolution was 50 m × 50 m in the Greater Boston Area. The algorithms used ground monitoring data collected from 987 monitoring sites across the contiguous U.S., satellite-derived measurements (available through the Google Earth Engine), chemical transport model simulations, meteorological conditions, and land-use data (e.g., traffic counts, distance to OpenStreetMap features), between 2000–2018. The predicted PMCs included Bromine (Br), Calcium (Ca), Copper (Cu), elemental carbon (EC), Iron (Fe), Lead (Pb), Nickel (Ni), nitrate (NO_3_^−^), organic carbon (OC), Potassium (K), Silicon (Si), sulfate (SO_4_^2−^), Vanadium (V), and Zinc (Zn). Excellent model performance was achieved with out of sample validation R^2^ for individual PMCs ranging from 0.821 (Br) to 0.975 (SO_4_^2−^). We further matched the annual average PMCs data with each residential address based on the grid cell centroid closest to the address and year at the time of DNA samples’ collection in the study population. Additionally, we predicted daily PM_2.5_ mass concentrations between 2000–2016, using an ensemble model at a resolution of 1 km × 1 km (R^2^ = 0.86). We then calculated the annual PM_2.5_ concentrations based on the daily data across that year [46]. The National Human Activity Pattern Survey in the U.S. reported that U.S. adults spent 69% of their time at home and 8% of the time immediately outside their home [47]. Given the age range of our study population, the time that they stayed at or near their residence was probably even longer. It is reasonable to use residential air pollution to capture the exposure.

### Source apportionment using positive matrix factorization

We used positive matrix factorization (PMF, version 5.0) analysis to apportion the measured PMCs to realizable sources [48]. PMF was developed by Paatero and Tapper [49] and has been widely used for sources apportionment studies [17, 50–52]. It requires two input files: the measured concentrations of the species and the estimated uncertainty of the concentration [48]. Similarly to cluster analysis, a correlation between species indicates a common factor which can represent a source category [48]. We tested for possible source numbers of 4,5, and 6. For each possible source number, 100 base runs were conducted to obtain the best factorization fit over all runs that achieved the minimization of weighted residual error for the linear fitting of a multivariate system of variables. We then selected the idea number of sources based on not only the minimization of weighted residual error but also the realistic scenario of component sources [50].

### DNAm measures

DNA samples were extracted using the IQAamp DNA Blood Kit (Qiagen, CA, U.S.) from the buffy coat of the whole food collected between 1999 and 2013 (We dropped samples in 1999 in this study because there were no predicted PMCs in that year). We measured DNAm using Illumina Infinium Human Methylation450K BeadChip (450 K; Illumina Inc., San Diego, CA, U.S.), which includes ~ 485,000 CpG sites. Based on a two-stage age-stratified algorithm, we randomized the samples across plates and chips to minimize batch effects [34]. As described previously, we preprocessed DNAm data via the *ewastools* package in Github [45]. We dropped low-quality samples [53] and corrected dye-bias using a regression on the logarithm of internal control probes [54]. We elaborated on the steps for probes cleaning previously [45]. In total, we included 360,272 high-quality probes remote from SNPs in this study.

We normalized DNAm data by controlling for the normalization factors in the outcome regression instead of using other commonly used approaches, such as beta-mixture quantile normalization [55]. This normalization approach ensures a better adjustment for batch effects as their impact often varies across probes, and we have applied it previously [45, 56, 57]. The normalization factors included five experimental covariates (i.e., Non-polymorphic Red, Specificity I Red, Bisulfite Conversion I Red, Bisulfite Conversion II, Extension Red) [58]. DNAm level was expressed as the ratio of methylated cytosines over the sum of the methylated and unmethylated cytosines at each CpG location and then multiplied by 100 (mean %5-methylcytosine, i.e., %5-mC). Thus, the DNAm level ranged from 0- to 100%5-mC.

### Statistical analyses

We examined EWAS of annual exposure to PMCs/sources at three levels: single CpG site, regional, and pathway analyses.

#### Single CpG analyses

We performed traditional EWAS at single CpG level to identify statistically significantly differentially methylated probes (DMPs) by PMCs/sources. As described previously [45], we used median regression to analyze associations between exposures and DNAm because median regression has no assumption on the distribution of dependent variables (i.e., DNAm in this study) [59]. In addition, we applied median regression for longitudinal data using the Koenker et al. method [60] because ~ 60% of the participants had repeated DNAm measures. It allowed us to model fixed-effects and correlated random-intercepts for each subject and use bootstrap for statistical inference. For PMCs, we investigated the effects of each PMC one a time with PM_2.5_ mass in the model to control for other particle components; for sources, we investigated the effects of each source with other three sources and PM_2.5_ mass in the model. In all regression analyses, we controlled for the following covariates a priori based on the relevant literature [34, 57]: chronological age (years), years of education, smoking status (ever/never), cigarette pack-years, alcohol consumption (< 2 or ≥ 2 drinks/day), body mass index (BMI, kg/m^2^), the estimated cell type compositions (CD4 + T lymphocytes, CD8 + T lymphocytes, natural killer cells, B cells, and monocytes) by the Houseman et al. method [61], technical factors such as batch effects and five normalization factors, and ambient annual mean temperature and relative humidity from gridMET [62]. The model for PMC is shown in Eq. (1) and the model for sources is shown in Eq. (2).

1 $${\mathrm{M}}_{ij}= {\beta }_{0}+ {\beta }_{1} \times {PMC}_{ij}+ {\beta }_{2} \times {PM}_{2.5ij}+\dots + {\beta }_{n} \times {X}_{ij}+ {\omega }_{i}+ {\varepsilon }_{ij}$$

2 $${\mathrm{M}}_{ij}= {\varphi }_{0}+ {\varphi }_{1} \times {Source1}_{ij}+ {\varphi }_{2} \times {Source2}_{ij}+\dots + {\varphi }_{m} \times {Sourcem}_{ij}+{\varphi }_{m+1} \times {PM}_{2.5ij}+\dots + {\varphi }_{n} \times {X}_{ij}+ {\theta }_{i}+ {\delta }_{ij}$$

, where M_*ij*_ is the median of DNAm level for subject _i_ at visit _j_. PMC_ij_ in Eq. (1) is the annual average of its concentration. Sourcem_ij_ in Eq. (2) is the annual concentration of the m^th^ (4 ≤ m ≤ 6) source for subject _i_ at visit _j_. (We determined the value of m as described in 2.3 Source appointment using positive matrix factorization.) PM_2.5ij_ are the annual average for subject _i_ at visit _j_. X_ij_ are the covariates that we listed above. ${\omega }_{i}$ in Eq. (1) and ${\theta }_{i}$ in Eq. (2) are the random intercepts for participant _i._ and ${\varepsilon }_{ij}$ in Eq. (1) and in Eq. (2) are the residuals. In order to account for the possible selection bias that healthier men were more likely to return for subsequent exams, we used inverse probability weighting [63]. It calculated the probability of having a subsequent visit given chronological age, education, BMI, blood pressure, smoking status, cigarette pack years, alcohol consumption, C-reactive protein, asthma, chronic bronchitis, and emphysema at previous visit, using logistic regression. We reported our results as the median difference in DNAm (%5-mC) per one interquartile range (IQR) increase in PMCs and its sources after annual exposure.

To account for the multiple testing in the context of the high correlation among CpG sites, we needed a method to consider that correlation, which reduces the effective number of independent tests. Following related work this area [64, 65], we used the “number of independent degrees of freedom” approach. Specifically, we used principal component analysis to project the probes to fewer dimensions. Fig. S1 showed the scree plot and cumulative scree plot that explained more than 95% of the variation of the DNAm data in this study. Thus, we set the number of independent degrees of freedom to be the number of components that explained 95% of the variation of the DNAm data (see Fig. S1). We then obtained the Bonferroni threshold for statistical significance of each estimate by dividing 0.05 by the independent degrees of freedom (i.e., PC-correction; *p*-value < 0.05/908/14 (3.93 × 10^–6^) for PMCs; *p*-value < 0.05/908/m for sources (4 ≤ m ≤ 6)).

#### Regional and pathway analyses

Single CpG may weakly associated with PMCs and its sources, and be difficult to identify. A region containing multiple DMPs that are functionally correlated may have more biological implications [66]. Thus, we investigated statistically significantly differentially methylated regions (DMRs) in relation to the exposures using the *comb-p* function from the *ENmix* package in R Bioconductor [67] because the *comb-p* tool has the best sensitivity and highest control of false-positive rate compared to the other DMR tools [68]. We defined a significant DMR as one with three or more probes within kilobase pair and its Sidak *p*-value < 0.05 [45].

In addition, we used the Ingenuity Pathway Analysis (IPA) database (QIAGEN Inc.) to identify significantly enriched gene pathways in the top ranked 100 CpGs that were associated with each PMC/source. We calculated permutation *p*-values based on the results of 10,000 random shuffles of association *p*-values for the CpGs on the 450 K array [69]. We defined significant pathways if *p*-value < 0.05 and gene set contains ≥ 3 genes with top ranked probes [45].

#### Sensitivity analyses

To check the robustness of our results, we conducted sensitivity analyses. In the main analysis, we only accounted for the selection bias due to healthier men being more likely to return for the subsequent exams. In the sensitivity analyses, we then further controlled for mortality that occurred prior to year 2000. We applied inverse probability weighting [63] via logistic regression to calculate the probability of death given the same factors that we mentioned above. We then multiplied this inverse probability weight with the one in the main analyses (for the probability of a subsequent visit). Thus, the visits in this study were representative of the original population. We compared the effects sizes and *p*-values of the top 5 probes for each source from the main analyses with the ones from the sensitivity analyses.

## Results

### Population description

We included 669 men with 1,178 visits. The summary characteristics of the study subjects are shown in Table 1. In this present study, almost sixty percent of the participants had more than one visit. The participants were older men with a mean age [standard deviation (SD)] of 74 (7) and 75 (7) at the first and all visits, respectively.

**Table 1:** Characteristics of elderly white men from the Normative Aging Study, 2000–2013

Variables	First visit (*N* = 669)	Second visit (*N* = 387)	Third visit (*N* = 121)	Fourth visit (*N* = 1)	All visits (*N* = 1,178)
Age, Mean ± SD	74 ± 7	76 ± 7	80 ± 6	76 ± NA	75 ± 7
BMI (kg/m^2^), Mean ± SD	28.10 ± 4.07	27.94 ± 4.33	27.84 ± 4.26	24.68 ± NA	28.02 ± 4.18
Years of education, Mean ± SD	15.07 ± 3.01	15.29 ± 3.08	15.84 ± 3.07	13.00 ± NA	15.22 ± 3.05
Smoking status, n (%)					
Never	205 (30.64%)	118 (30.49%)	37 (30.58%)	0 (0%)	360 (30.56%)
Ever	464 (69.36%)	269 (69.51%)	84 (69.42%)	1 (100%)	818 (69.44%)
Pack-years, Mean ± SD	25.43 ± 21.38	20.48 ± 23.86	20.01 ± 23.52	15.00 ± NA	20.94 ± 24.71
Alcohol consumption (drinks/day), n (%)					
˂ 2	540 (80.72%)	318 (82.17%)	100 (82.64%)	0 (0%)	958 (81.32%)
≥ 2	129 (19.28%)	69 (17.83%)	21 (17.36%)	1 (100%)	220 (18.68%)
Estimated cell type (%), Mean ± SD					
Granulocytes	57.51 ± 9.24	58.83 ± 10.07	61.91 ± 8.66	63.35 ± NA	58.40 ± 9.55
Monocytes	10.75 ± 2.74	10.16 ± 3.08	10.31 ± 2.60	9.49 ± NA	10.51 ± 2.85
B cells	1.63 ± 2.75	1.20 ± 2.37	1.11 ± 2.44	0.62 ± NA	1.43 ± 2.60
CD4 + T lymphocytes	8.42 ± 4.39	8.31 ± 4.76	7.37 ± 3.40	15.53 ± NA	8.28 ± 4.43
CD8 + T lymphocytes	8.70 ± 3.15	8.52 ± 3.17	7.40 ± 2.83	6.95 ± NA	8.51 ± 3.14
Natural killer cells	7.11 ± 3.86	7.57 ± 4.35	7.42 ± 4.11	4.19 ± NA	7.29 ± 4.05

Abbreviations: *BMI* body mass index, *SD* standard deviation

### Concentrations of PM_2.5_ and 14 PMCs

Table 2 presents the summary statistics of annual PM_2.5_ and its PMCs during the study period (2000–2013). The mean (SD) concentration of annual PM_2.5_ mass concentration was 9.75 (1.80) µg/m^3^, with an IQR of 2.39 µg/m^3^. Among the investigated PMCs, SO_4_^2−^ accounted for the largest proportion of PM_2.5_ total mass (30.5%), followed by OC (19.4%). The annual average of metal components such as Pb and Fe is 3.22 (SD 1.09) ng/m^3^ and 50.84 (15.60) ng/m^3^, respectively. We reported the correlation coefficients among PM_2.5_ mass and 14 PMCs in Table S1 (see Table S1 in the supplementary material). The correlation coefficients ranged from 0.00 for Cu and K to 0.80 for Cu and Fe. K is the components that had the lowest correlation with other PMCs.

**Table 2:** Distribution of annual PM_2.5_ and its components in the Normative Aging Study, 2000–2013

Particles	Min	Mean (SD)	Median (Q1, Q3)	Max	IQR
PM_2.5_ (µg/m^3^)	4.16	9.75 (1.80)	9.78 (8.60, 11.00)	16.02	2.39
EC (µg/m^3^)	0.18	0.48 (0.12)	0.47 (0.41, 0.53)	1.19	0.12
OC (µg/m^3^)	0.86	1.89 (0.41)	1.82 (1.59, 2.16)	3.61	0.57
NO_3_^−^ (µg/m^3^)	0.23	0.89 (0.22)	0.88 (0.72, 1.04)	1.53	0.32
SO_4_^2−^ (µg/m^3^)	0.81	2.97 (0.90)	3.06 (2.63, 3.24)	6.29	0.61
Br (ng/m^3^)	1.58	2.69 (0.31)	2.70 (2.52, 2.90)	3.60	0.38
Ca (ng/m^3^)	11.82	24.60 (4.87)	24.09 (21.52, 26.80)	46.25	5.29
Cu (ng/m^3^)	0.49	2.58 (0.98)	2.60 (1.86, 3.19)	6.97	1.33
Fe (ng/m^3^)	15.28	50.84 (15.60)	51.65 (39.70, 61.03)	111.47	21.34
K (ng/m^3^)	30.70	47.69 (3.71)	47.32 (45.44, 49.89)	66.75	4.45
Ni (ng/m^3^)	0.06	1.39 (0.61)	1.32 (0.90, 1.79)	3.43	0.89
Pb (ng/m^3^)	1.09	3.22 (1.09)	3.32 (2.25, 4.01)	5.45	1.76
Si (ng/m^3^)	27.59	64.64 (19.47)	63.92 (47.17, 80.40)	113.92	33.23
V (ng/m^3^)	0.19	2.54 (1.18)	2.61 (1.70, 3.32)	7.44	1.62
Zn (ng/m^3^)	3.21	10.27 (2.54)	10.10 (8.54, 12.34)	18.22	3.80
Temperature (°C)	0.04	10.31 (2.77)	11.02 (9.27, 12.18)	21.62	2.91
Relative humidity (%)	32.89	62.17 (9.02)	66.17 (56.39, 68.71)	81.72	12.32

Abbreviations: *Br* Bromine, *Ca* Calcium, *Cu* copper, *EC* elemental carbonM, *Fe* iron, *Pb* lead, *Ni* nickel, *NO*^*3−*^ nitrate, *OC* organic carbon, *K* potassium, *Si* silicon, *SO*_*4*_^*2−*^, sulfate, *V* vanadium, *Zn* zinc

### Sources from PMF analysis

**Fig. 1:**
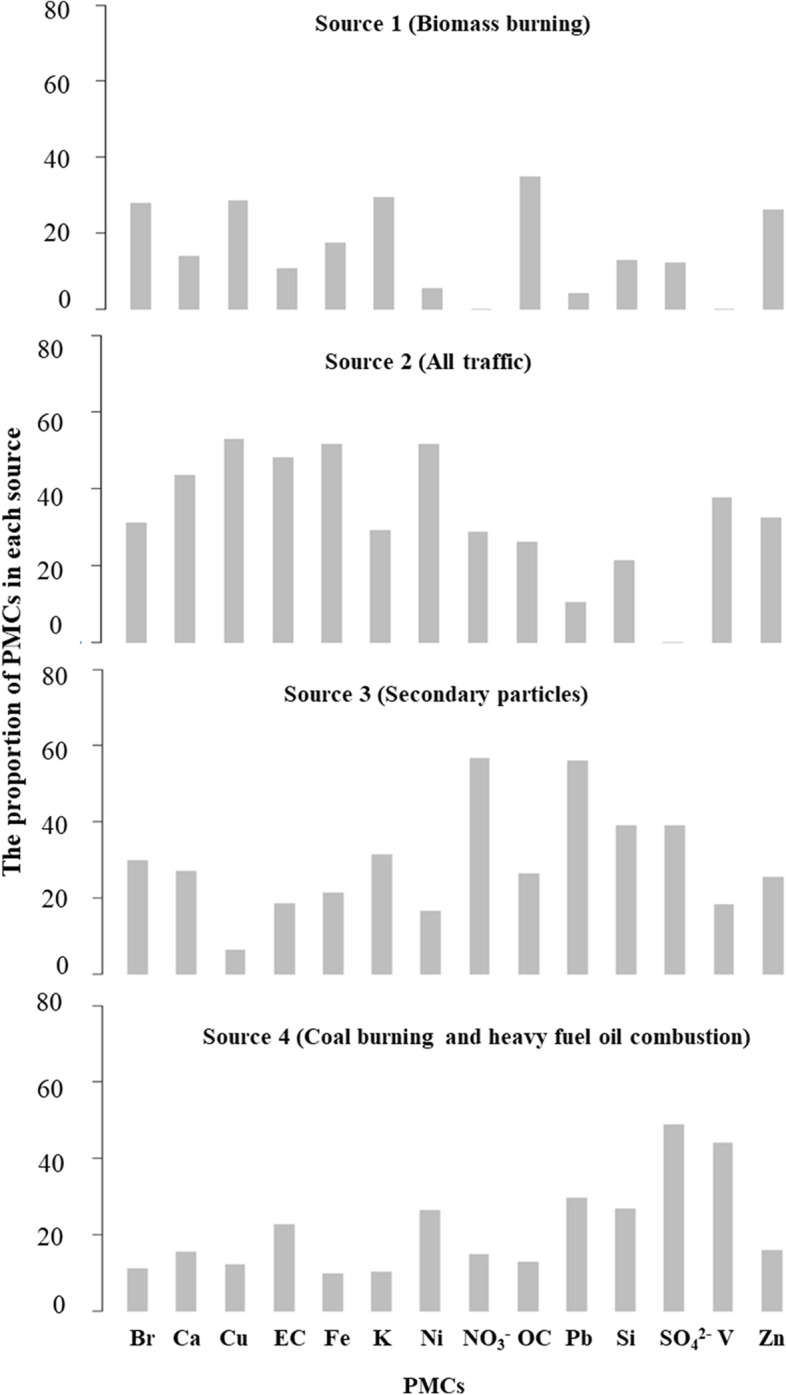
The proportion of PMCs in each source. Abbreviations: PMC, particulate matter components; Br, bromine; Ca, calcium; Cu, copper; EC, element carbon; Fe, iron; K, potassium; Ni, nickel; NO_3_-, nitrate; OC, organic carbon; Pb, lead; Si, silicon; SO_4_^2^-, sulfate; V, vanadium; Zn, zinc

Based on an evaluation of three PMF models with the number of sources equal to 4, 5, and 6, we chose 4 as it provided the most feasible source results. The source profiles and the distributions of 14 PMCs are presented in Fig. 1. The four sources included biomass burning (source 1), all traffic (source 2), secondary particles (source 3), and coal burning and heavy fuel oil combustion (source 4).

### EWAS of PMCs and sources

#### Significant probes

In the site-by-site analyses, we observed multiple significant DMPs for the 14 PMCs and 4 sources (see Table 3). For example, we found 7 significant DMPs associated with SO_4_^2−^, 11 with K, 10 with source 1, and 8 with source 2. We presented the significant DMPs associated with PMCs ranked by *p*-values with their annotated genes in Tables S2; we showed the significant DMPs associated with sources ranked by *p*-values with their annotated genes in Tables S3. We also presented the Manhattan plots and the quantile–quantile plots with the estimated genomic inflation factor for each exposure (Fig. S2) and source (Fig. S3).We compared the significant DMPs by the 4 sources and found a few common probes across sources: source 1 had a common probe with source 2 (cg15911114), 6 common probes with source 3 (cg09852920, cg04660698, cg25277509, cg01252659, cg01733795, cg10692118), and 4 common probes with source 4 (cg09852920, cg25277509, cg01252659, cg10692118); source 3 and source 4 had 12 overlapping probes (cg09852920, cg16756998, cg21675770, cg25277509, cg25631650, cg10692118, cg05524450, cg21468420, cg01252659, cg17367077, cg08753391, cg05970846). Additionally, sources 1, 3, and 4 had 4 common probes (cg09852920, cg25277509, cg01252659, cg10692118). We also compared the significant DMPs by 14 PMCs (see supplementary material). There were many DMPs that were not similar across sources or PMCs.

**Table 3:** The number of significantly differentially methylated probes, regions, and pathways from annual exposure to 14 PM_2.5_ components and 4 sources

Exposures	DMPs	DMRs	Pathways
PM_2.5_ components			
Br	58	34	2
Ca	1	5	16
Cu	13	10	3
EC	0	6	8
Fe	6	6	2
K	11	18	9
Ni	8	22	4
NO_3_^−^	15	3	46
OC	2	2	6
Pb	0	3	2
Si	10	1	7
SO_4_^2−^	7	3	3
V	5	11	27
Zn	7	10	8
Sources			
Source 1 (biomass burning)	10	0	8
Source 2 (all traffic)	8	5	0
Source 3 (secondary particles)	19	5	2
Source 4 (coal burning and heavy fuel oil combustion)	14	7	7

Abbreviations: *PM*_*2.5*_ fine particulate matter with an aerodynamic diameter ≤ 2.5 µm, *Br* bromine, *Ca* calcium, *Cu* copper, *EC* element carbon, *Fe* iron, *K* potassium, *Ni* nickel, *NO*_*3*_^*−*^ nitrate, *OC* organic carbon, *Pb* lead, *Si* silicon, *SO*_*4*_^*2−*^ sulfate, *V* vanadium, *Zn* zinc

### Significant regions and pathways

We identified multiple significant DMRs for PMCs and sources (see Table 3). For example, we observed 6 DMRs due to EC: chr19: 37,825,307–37825680, chr4: 57,773,149–57,773,309, chr12: 14,720,834–14721289, chr3: 48,694,451–48,694,674; chr14: 24,779,959–24,780,405; chr17: 3,704,494–3704622 and 5 DMRs due to source 3 (i.e., secondary particles): chr6: 29,594,830–29595662, chr 16: 8,806,531–8807044, chr6: 33,048,254–33048486; chr10: 32,216,031–32216391, chr11: 6,291,879–6,292,312. We presented the significant regions ranked by *p*-values for PMCs in Table S4 and for sources in Tables S5. We compared the significant DMRs and the annotated genes with 4 sources and found that sources 3 and 4 had 5 common DMRs (chr16: 8,806,531–8807044, chr6: 29,594,830–29595662, chr10: 32,216,031–32216391, chr6: 33,048,254–33048286, chr11: 6,291,879–6,292,312) and 5 common annotated genes (*ABAT*, *GABBR1*, *ARHGAP12*, *HLA-DPB1*, *CCKBR*). We also compared the significant DMRs by 14 PMCs (see supplementary material).

In the pathway analyses, we found multiple significant pathways in relation to PMCs and sources (see Table 3). For example, we found 8 pathways for EC: role of NFAT in cardiac hypertrophy, telomerase signaling, tight junction signaling, cellular effects of sildenafil, osteoarthritis pathway, Wnt/ β-catenin signaling, cyclins and cell cycle regulation, axonal guidance signaling and 2 pathways for source 3: PTEN signaling and ILK signaling.

We showed the significant pathways ranked by *p*-values for PMCs in Tables S6 and for sources in Tables S7. We compared the significant pathways of the 4 sources: the two significant pathways of source 3 and the seven significant pathways of source 4 were all included in the eight pathways of source 1 (e.g., PTEN signaling).

### Sensitivity analyses

We extracted the top 5 probes from the EWAS of 4 sources in the main analyses and compared their effect sizes and *p*-values with that from the sensitivity analyses. The effect sizes in the sensitivity analyses for all four sources were almost the same as in the main analyses (see Fig. S4).

## Discussion

To our knowledge, this is the first EWAS of PMCs/sources using high-resolution air pollution models with 50 m × 50 m resolution. We identified multiple DMPs, DMRs, and pathways associated with both PMCs and PM_2.5_ sources. Moreover, the identified DMPs, DMRs, and pathways were different across PMCs and sources. For example, the significant pathways suggest that the source 1 (i.e., biomass burning) was related to CVD cancer, diabetes, inflammation, Alzheimer’s disease (AD), and oxidative stress, whereas the source 3 (i.e., secondary particles) was annotated to CVD and cancer.

In terms of the significant pathways associated with PM_2.5_ sources, source 1 (i.e., biomass burning) and source 4 (i.e., coal burning and heavy fuel oil combustion) were associated with almost the same pathways, such as cancer [70, 71], inflammation [72], CVD [73], AD [74, 75], and diabetes [76]. For example, the mTOR signaling pathway is related to cancer [70], and AD [74]; IL-8 signaling is associated with inflammation [72]; insulin receptor signaling is linked to diabetes [76]*.* The two pathways associated with the source 3 (i.e., secondary air pollution) were linked with cancer [71] and CVD [73]*.* For example, ILK signaling has been associated with the human heart [73]. There were no significant pathways that were associated with source 2 (i.e., all traffic). These findings suggest that biomass burning, coal burning, and heavy fuel oil combustion were the most impactful PM_2.5_ sources with respect to DNAm; all traffic had the least impact on DNAm in the Greater Boston Area.

We further discuss the significant pathways associated with individual PMCs based on their proportions across four sources. The highest proportion of OC was from the source 1 (i.e., biomass burning) and it was associated with pathways of cancer [77] and inflammation [72]. For example, RhoGDI signaling has been shown to mediate cancer progression [77]. Source 3 (i.e., secondary air pollution) had the highest percentage of NO_3_^−^. The pathways linked with NO_3_^−^ were involved with CVD [78–85], cancer [86–88], inflammation [72, 89, 90], obesity [91], depression [92], and schizophrenia [93]. For example, cAMP-mediated signaling is typically involved in the regulation of heart function [78]; CXCR4 signaling contributes to tumor growth and invasion [86]. Source 4 (i.e., coal burning and heavy fuel oil combustion) accounted for the highest V, which was associated with pathways in immune system [94], cancer [77, 88, 95, 96], and AD [97]. Although source 2 (i.e., all traffic) was not associated with any pathways, its main components Ca, EC, and K were related with a few pathways, including cancer [70].

While studies of PM_2.5_ have been done, DMPs and pathways associated with only certain PMCs would be less likely to be detected in studies of that composite exposure. Hence this study, with repeated measures and high-resolution exposure to many components, is an important advance. Until recently, only a few epidemiological studies have linked long-term exposure to PMCs/sources with the pathways that we identified in this study. [98, 99]. For example, Ostro et al. found that long-term exposure to high-sulfur fuel combustion and the secondary particle NO_3_^−^ was associated with CVD mortality in a longitudinal study [99]. This is consistent with our findings of pathways associated with secondary particles and coal burning.

We did not observe any significant associations with long-term exposure to source 2 (i.e., all traffic) and pathways. However, a few epidemiological studies found that exposure to traffic-related exposures were related cellular immunity, cardiovascular and neurological systems development, inflammation [28, 100, 101] among the DNA methylation features. For example, Eze et al. performed EWAS of transportation air pollution exposures. Their agnostic functional networks found cellular immunity, gene expression, cell growth/proliferation, cardiovascular, auditory, embryonic, and neurological systems pathways [28]. The inconsistency may be attributable to the agents in the traffic sources. In our study, the traffic source is mostly consistent of Cu, Fe, EC, and Ca, whereas other studies use nitrogen dioxide and PM_2.5_ as the main agents for traffic-related toxicity [102–104].

To date, only one study examined the associations between long-term exposure to PMCs and DNAm in an epigenome-wide scope [34], which was also conducted by our group (We did not perform an EWAS of sources in the previous study). This study used a central site for measurements of particle components. The number of significant DMPs (*N* = 29) and pathways (*N* = 9) was fewer in the previous study compared with this present one. We did not investigate the regions significantly associated with PMCs in our previous study, which observed a total of 29 DMPs (20 for Fe, 8 for Ni, and 1 for V) and 9 pathways (8 for Fe, 2 for Ni). In contrast, this present study totally identified 143 DMPs (among which 6 for Fe, 8 for Ni, and 5 for V) and 143 pathways (among which 2 for Fe, 4 for Ni, and 27 for V). We did not find any common DMPs, but a few overlapping pathways in the two studies, such as pathways in cancer by Ni. The different results in the two studies are mainly attributed to the data source of PMCs. The previous study estimated the concentrations of PMCs from monitors at a stationary site whereas the present study predicted the PMCs from high-resolution models with 50 m × 50 m.

In addition, we compared the significant DMPs in the present study with EWAS of long-term exposure to other air pollutants, such as PM2.5 mass [25, 27, 28, 105], smoking [106], coaling-burning [107], nitrogen oxides [27], sulfur oxide [108], and polycyclic aromatic hydrocarbons [109]. We found 8 overlapping DMPs for our components with DMPs previously identified for smoking [108], and 6 overlapping DMPs for sulfur oxide exposure [108], respectively. Specially, Joehanes et al. compared both current and past smokers with nonsmokers in DNAm using 16 cohorts and identified 18, 760 DMPs in current smokers and 2,623 DMPs in former smokers. Among the significant DMPs, 8 from current smokers (i.e., cg07450086, cg06644515, cg11436113, cg02324920, cg24807850, cg07197831, cg27134322, cg05661533) and 1 from former smokers (i.e., cg11436113, which was also identified in current smokers) were overlapped in this present study. These 8 DMPs were significantly associated with Fe, Zn, Ni, and K in our study. Among the mapped genes, the *SELENOT* gene is highly expressed in the cerebral globus pallidus and caudate nucleus in patients with Parkinson’s disease [110]. Choi et al. found a total of 6,733 DMPs were associated with prenatal exposure to sulfur oxide during the 3^rd^ trimester at age 2 [108], among which 6 DMPs were also identified in our study (i.e., cg09835867, cg01747792, cg05871607, cg07143898, cg25142954, cg05661533). These 6 DMPs were significantly associated with Si, Zn, and Br in our study. Among the mapped genes, high expression of *LPCAT1* gene plays an important role in breast cancer progression [111]. However, we did not find any common DMPs between our study and EWAS of PM_2.5_ [25, 27, 28, 107], nitrogen oxides [27], or polycyclic aromatic hydrocarbons [109]. This may reflect the heterogeneity in DMPs across different particle components.

This study has some limitations: 1) This study only included elderly white men, which limits the generalizability of the findings to other age groups, races, and sex. However, the studies that assess the modification effects on age, race, and sex in the associations between PMCs and DNAm are not well-established [112]. 2) We do not have data on gene expression; thus, we are not able to determine the regulation directions between DNAm and the coded protein. 3). We only measured DNAm in leukocytes, hence the identified pathways via IPA database (which is built based on multiple tissues) may not reflect all the relevant pathways.

On the other hand, our study has a number of important strengths. 1) This is the first EWAS to assess the associations between DNAm and PMCs/sources using high-resolution models (i.e., 50 m × 50 m). 2) The median regression that we used in this study does not require normally distributed residuals. 3) Repeated measurements of DNAm and PMCs provide a wide variation of the outcomes and exposures within-subject; thus, the statistical power is increased. 4) We analyzed EWAS of PMCs/sources at multiple dimensions: single CpG site, region, and pathway. It ensures us to fully elucidate the genes and pathways lined with the exposures.

## Conclusions

In summary, this EWAS of long-term exposure to PMCs/sources from high-resolution models indicates that the associations between DNAm and particles varies by the components and sources. PMCs with emission sources of biomass burning, coal burning, and heavy fuel oil combustion are the most harmful. More similar studies with diverse study populations from different areas, using DNAm from other tissues are needed, to enrich the present findings in the future.

### Supplementary Information

**Additional file 1.**

## Data Availability

The PM_2.5_ components concentration data are generated from the exposure models developed by Heresh Amini (heresh.amini@sund.ku.dk), and are available online at the SEDAC website. The methylation data are available at dbGAP, while the phenotype data are not available due to privacy restrictions. The meteorology data are available for download from the GridMET website.

## Abbreviations

Br: Bromine
Ca: Calcium
Cu: Copper
EC: Element carbon
EWAS: Epigenome-wide association analysis
Fe: Iron
IQR: Interquartile range
K: Potassium
Ni: Nickel
NO_3_^−^: Nitrate
OC: Organic carbon
Pb: Lead
PMC: Particulate matter components
PM_2.5_: Fine particulate matter
Si: Silicon
SO_4_^2^^−^: Sulfate
V: Vanadium
Zn: Zinc
