# Supplementary material for: Long-term exposure to ambient fine particulate components and leukocyte epigenome-wide DNA Methylation in older men: the Normative Aging Study
Source: Environ Health. 2023 Aug 7;22:54. doi: 10.1186/s12940-023-01007-5 (PMC10405403; doi:10.1186/s12940-023-01007-5)

**Supplementary Information**

**Long-term exposure to ambient fine particulate components and leukocyte epigenome-wide DNA Methylation in older men: the Normative Aging Study**

**Results**

**Significant probes**

We compared the significant DMPs by 14 PMCs and found that only Br had a few common probes with other PMCs: 3 common probes with Cu (cg27250362, cg03310453, cg08579070), 2 common probes with NO_3_^-^ (cg24229819, cg27637895), one with Si (cg09835867), and one with V (cg10209391).

### **Significant regions and pathways**

We compared the significant DMRs and the mapped genes with 14 PMCs and found that there were a few common DMRs and genes: one common DMR and gene from two PMCs: Br - OC (chr17: 17109239-17110121, *PLD6*), Ca - Fe (chr10: 133793442-133793735, *BNIP3*), Cu - V (chr20: 57427274-57427974, *GNAS*), NO_3_^-^ - SO_4_^2-^ (chr6: 88757302-88757704, *SPACA1*), Pb - Si (chr17: 33759484-33760528, *SLFN12*); one common DMR and gene from three PMCs: Ca - Ni - V (chr6: 32116538-32118812, *PRRT1*), Cu - Fe - Ni (chr6: 31846769-31847010, *SLC44A4*); one common DMR and gene from four PMCs: EC - Fe - Ni - Zn (chr17: 3704494-3704622, *ITGAE*); two common DMRs and genes from two PMCs: Ni - V (chr11: 46366419-46367101, *DGKZ*; chr5: 158524270-158524650, *EBF1*).

We also compared the significant pathways of the 14 PMCs and found some common pathways: e.g., Ca - K - NO_3_^-^ (AMPK signaling), Ca - NO_3_^-^ - Si (Thrombin signaling), Ca – Ni - NO_3_ ^-^ SO_4_^2-^ - V - Zn (protein kinase A signaling), Ca - NO_3_^-^ (PPARα/RXRα activation), Ca - NO_3_^-^ - OC (IL-8 signaling), Ca - K -V (role of osteoblasts osteoclasts and chondrocytes in rheumatoid arthritis), Ca - EC - NO_3_^-^ - V (role of NFAT in cardiac hypertrophy, K - NO_3_^-^ -V (CREB signaling).

**Figure S1.** Scree and cumulative scree plots for this study.

**Figure S2.** Manhattan and quantile-quantile plots with the estimated genomic inflation factor for each PM2.5 component in the main analysis.

**Figure S3.** Manhattan and quantile-quantile plots with the estimated genomic inflation factor for each source in the main analysis.

**Figure S4.** Effect size and 95% confidence intervals for the top 5 probes in the main analyses and sensitivity analyses.

**Table S1.** The Pearson correlation among PM_2.5_ and its 14 PMCs in the Normative Aging Study, 2000-2013.

**Table S2.** The significant DMPs ranked on *p*-value associated with one IQR increase in each PMC in the site-by-site analyses.

**Table S3.** The significant DMPs ranked on *p*-value associated with one IQR increase in each source in the site-by-site analyses.

**Table S4.** The significant DMRs ranked on *p*-value associated with one IQR increase in each PMC in the regional analyses.

**Table S5.** The significant DMRs ranked on *p*-value associated with one IQR increase in each source in the regional analyses.

**Table S6.** The significant pathways due to each PMC using the Ingenuity Pathway Analysis.

**Table S7.** The significant pathways due to each source using the Ingenuity Pathway Analysis.

**Figure S1:** Scree and cumulative scree plots for this study.


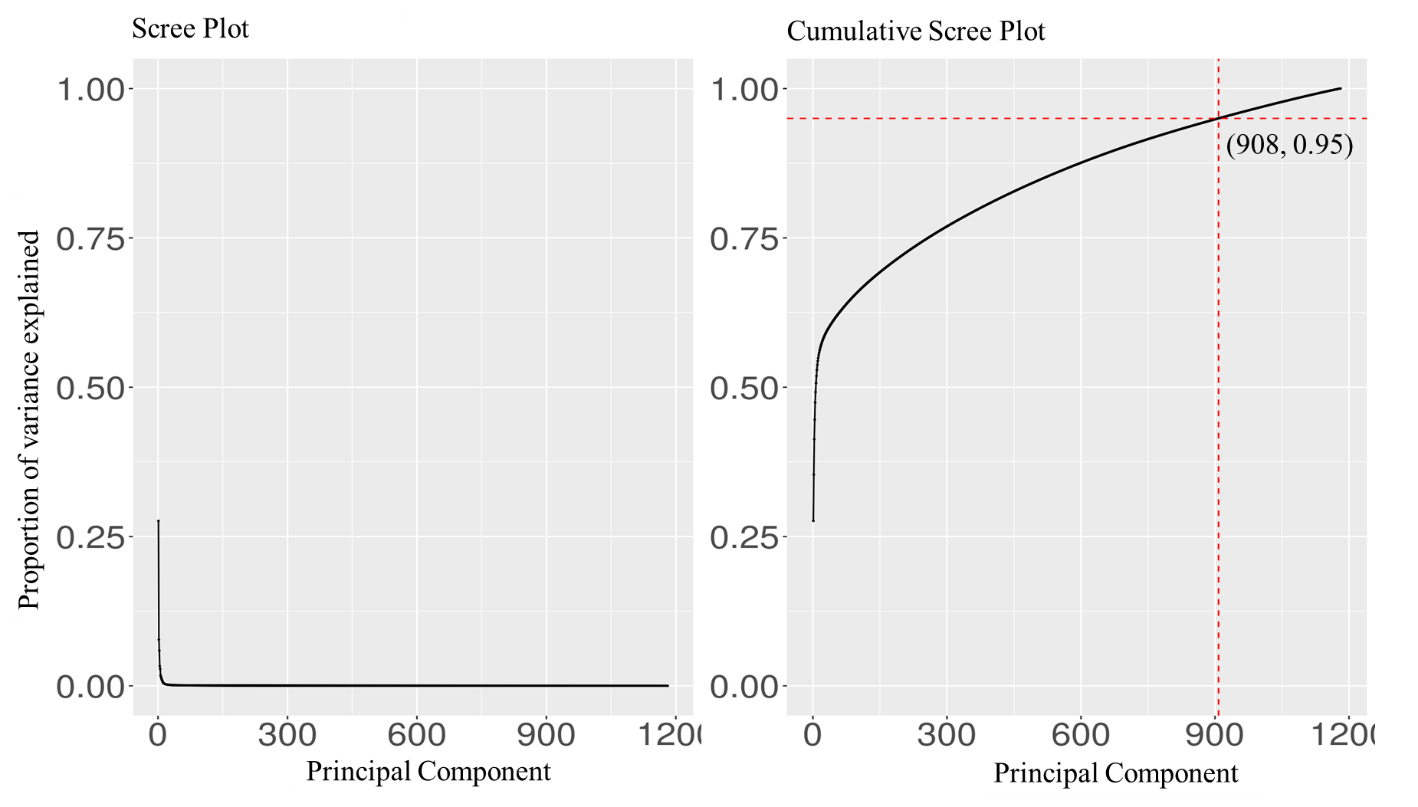


**Figure S2.** Manhattan and quantile-quantile plots with the estimated genomic inflation factor for each PM2.5 component in the main analysis.


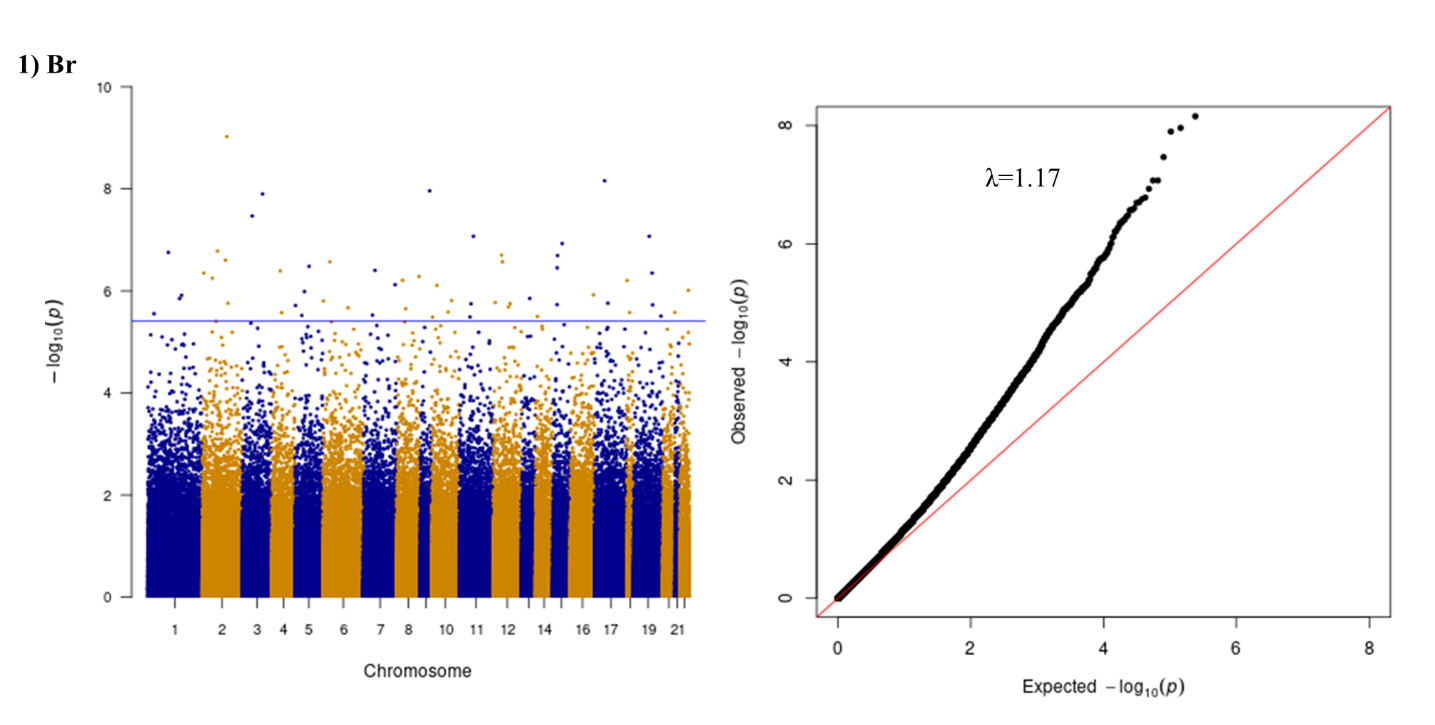


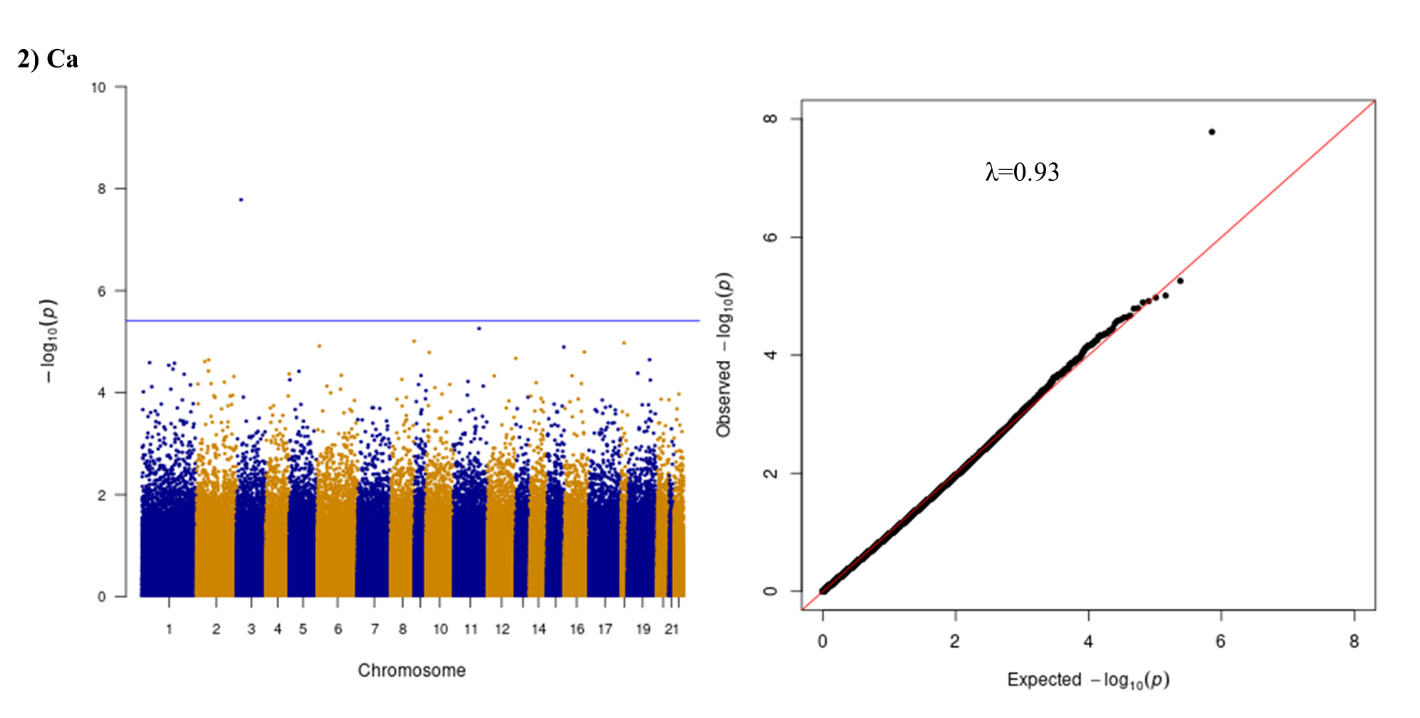


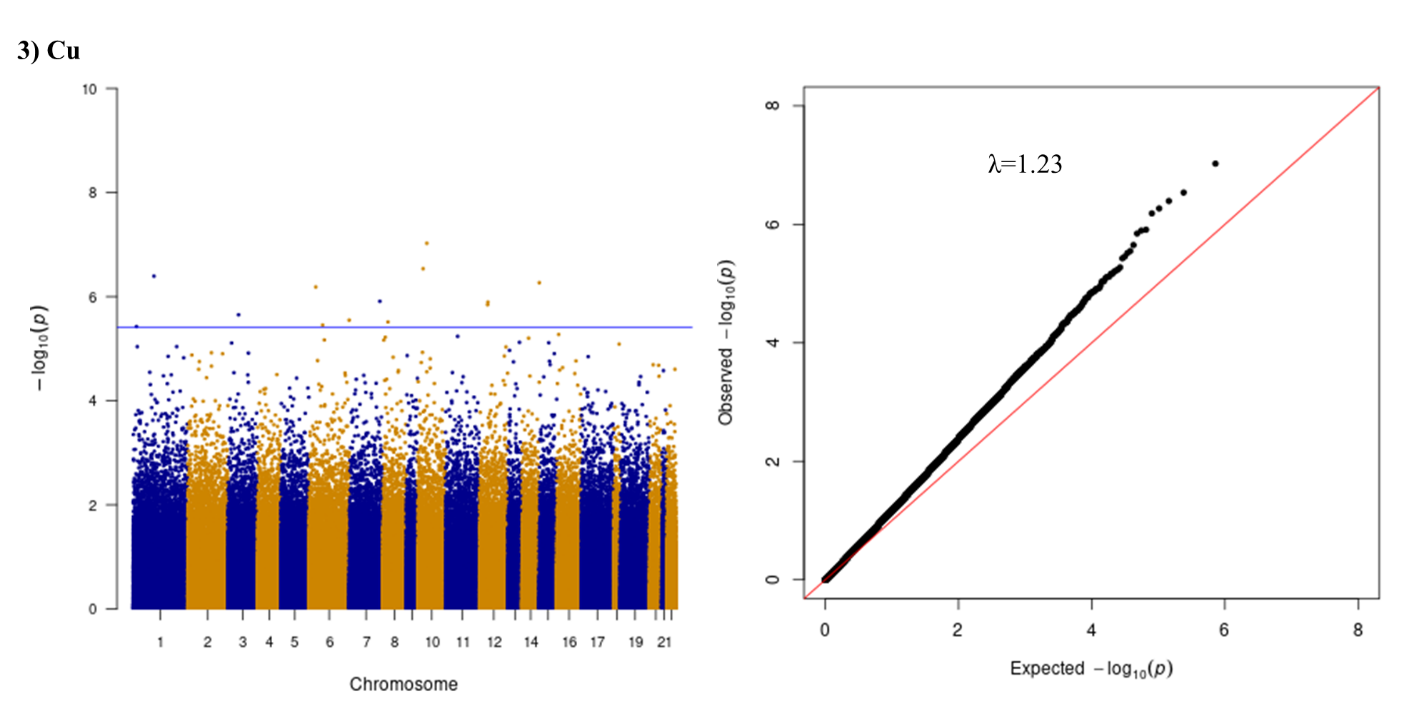


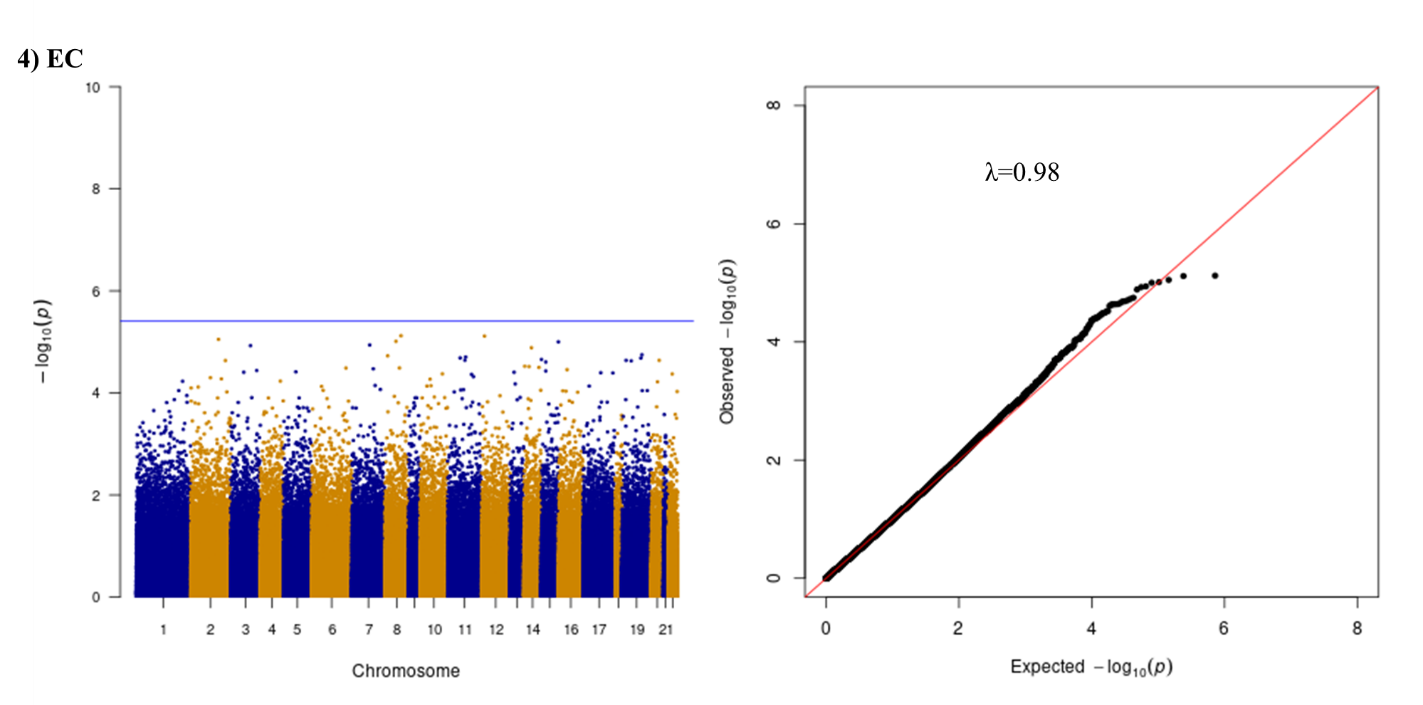


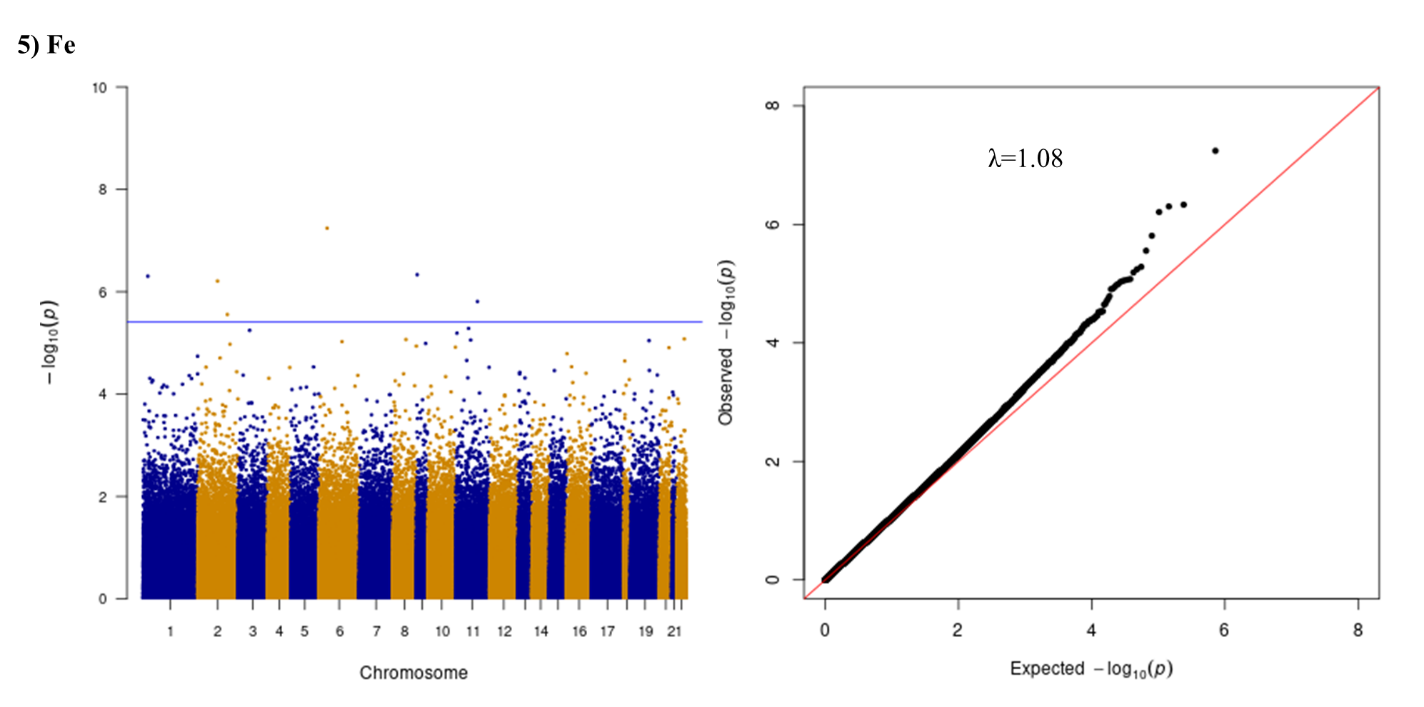


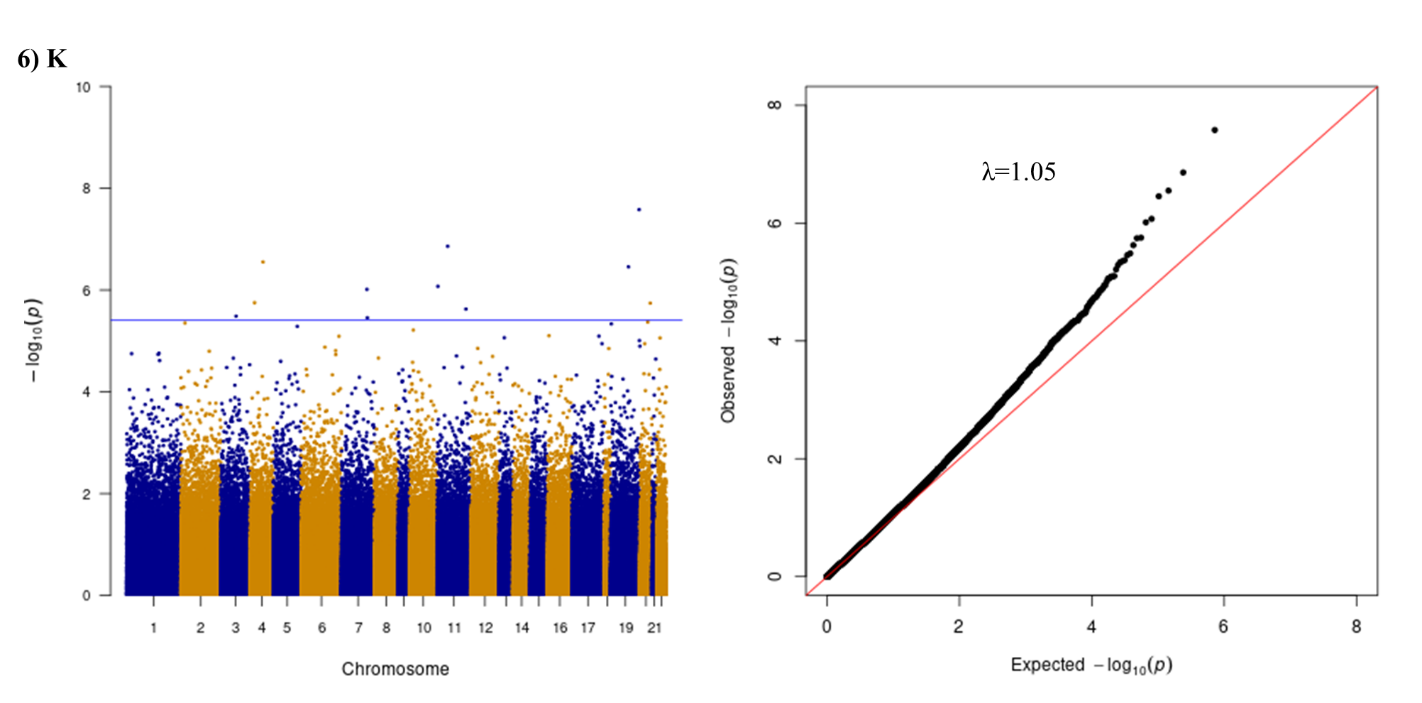


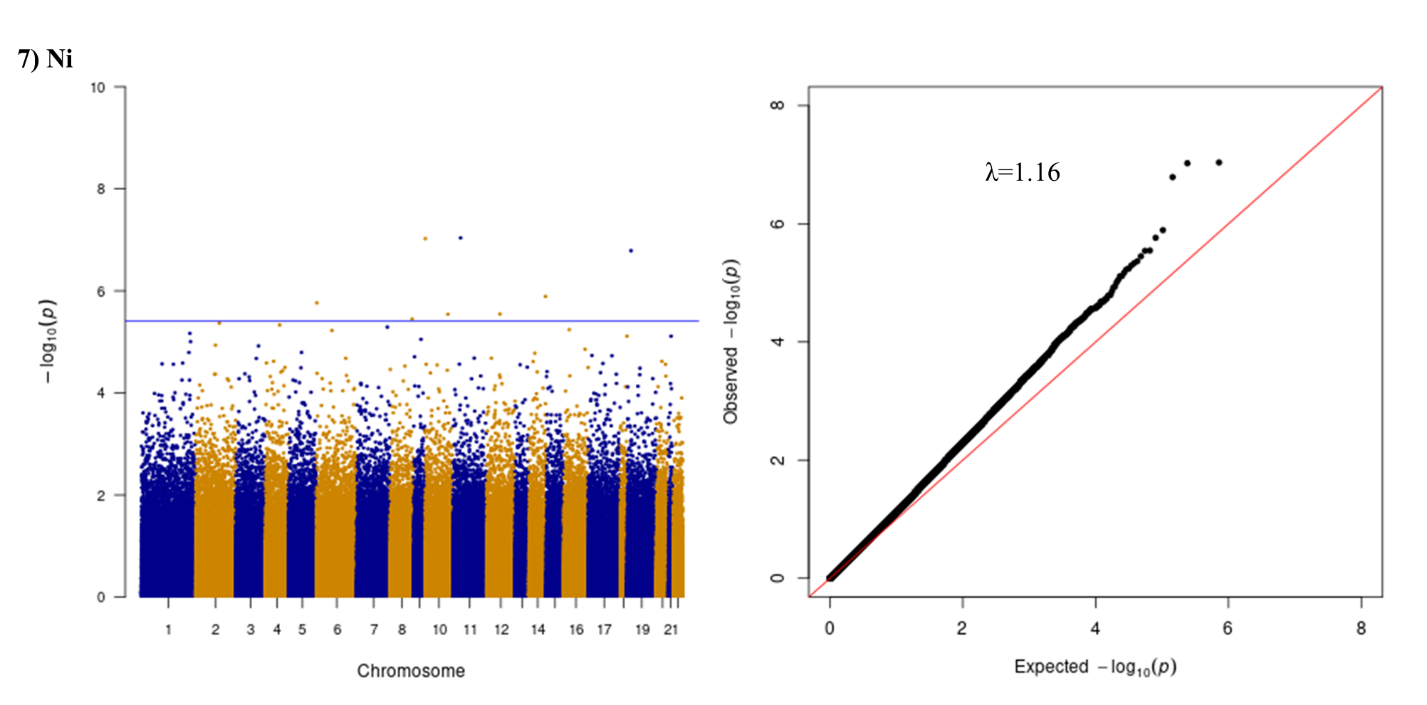


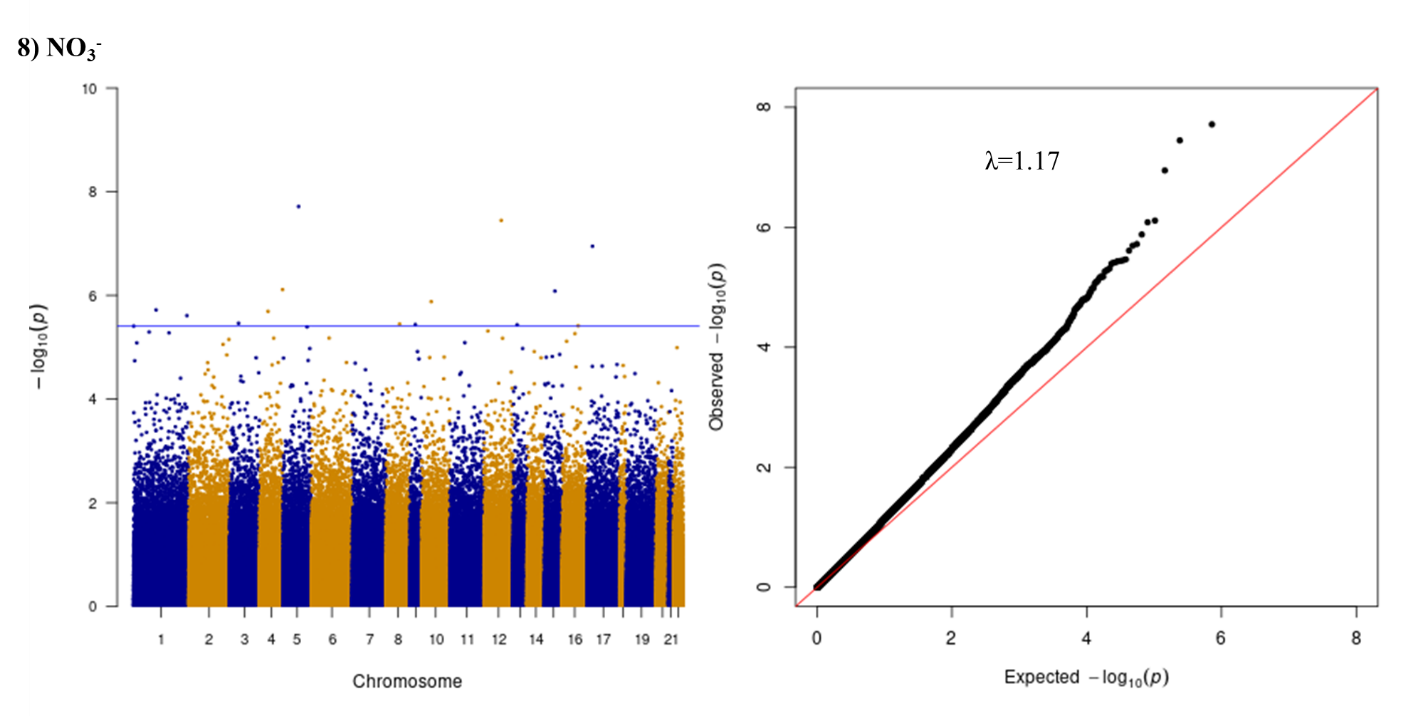


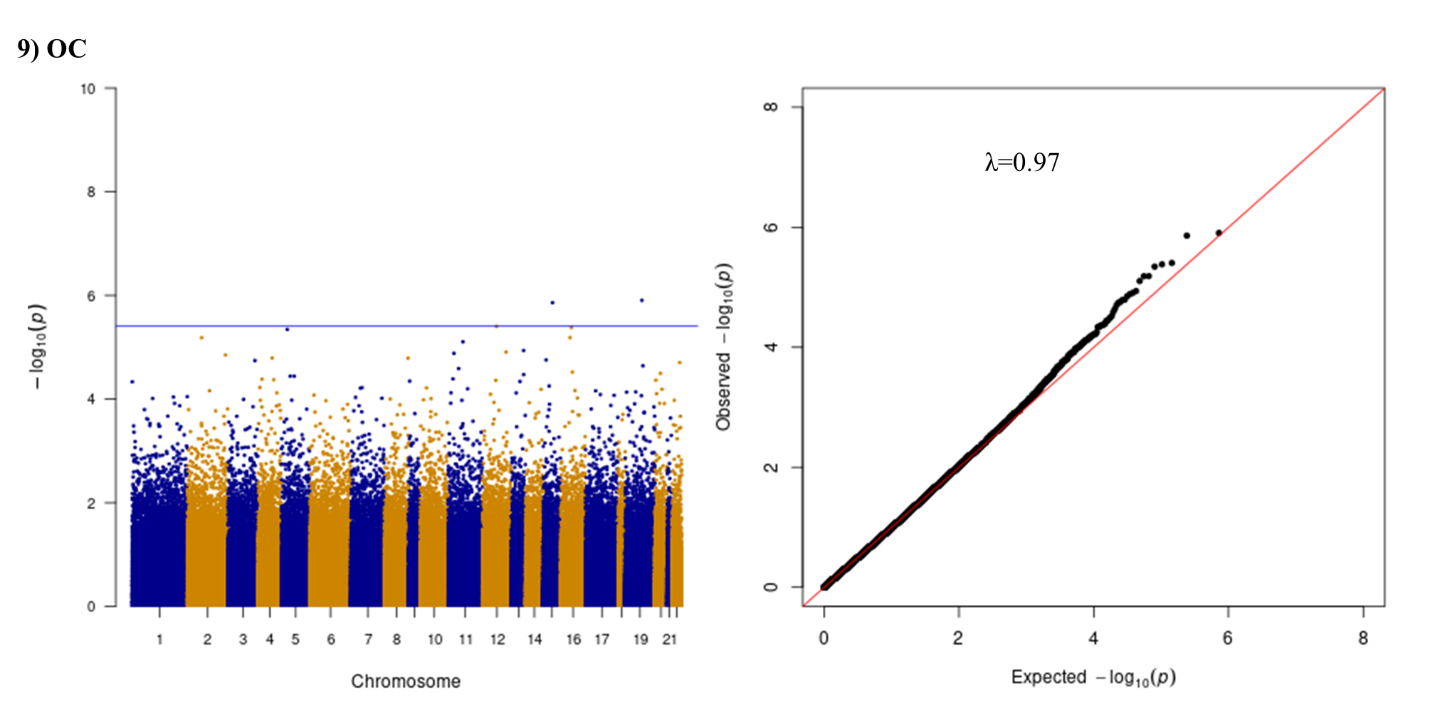


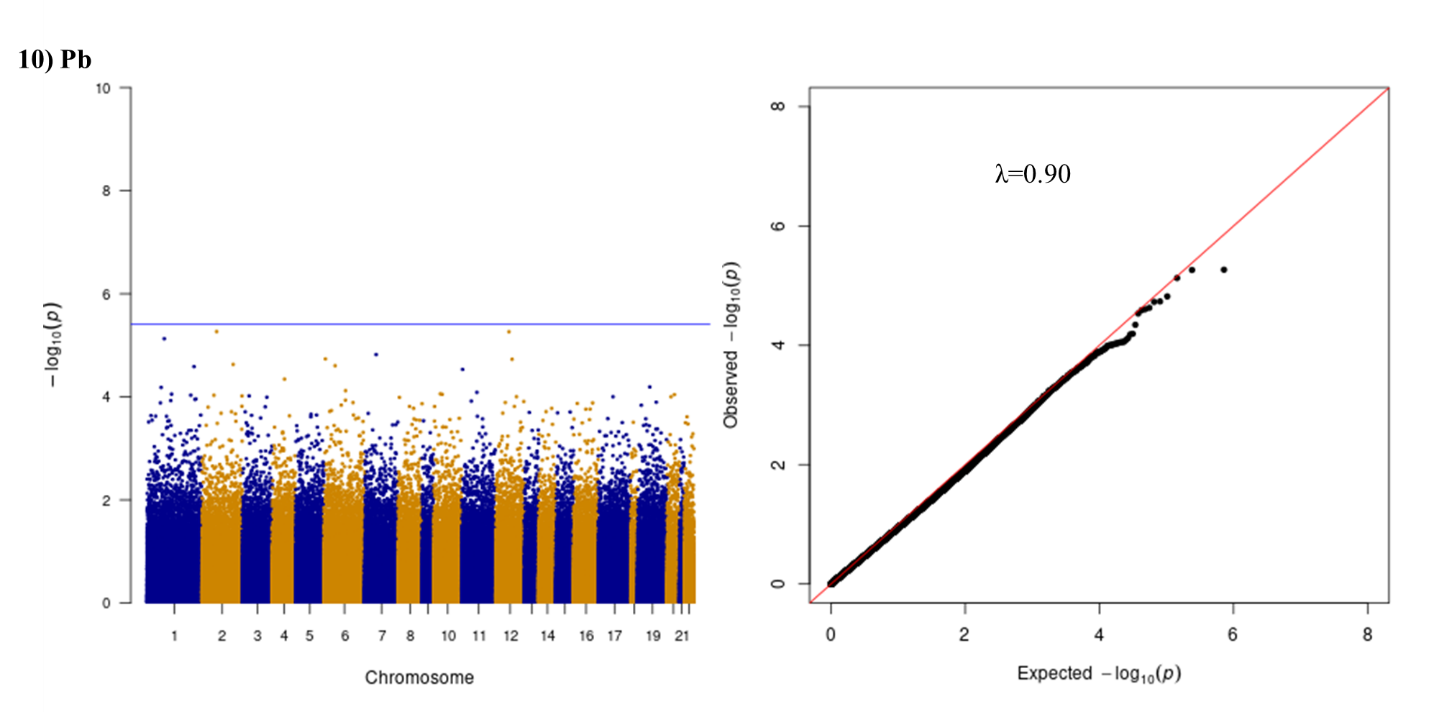


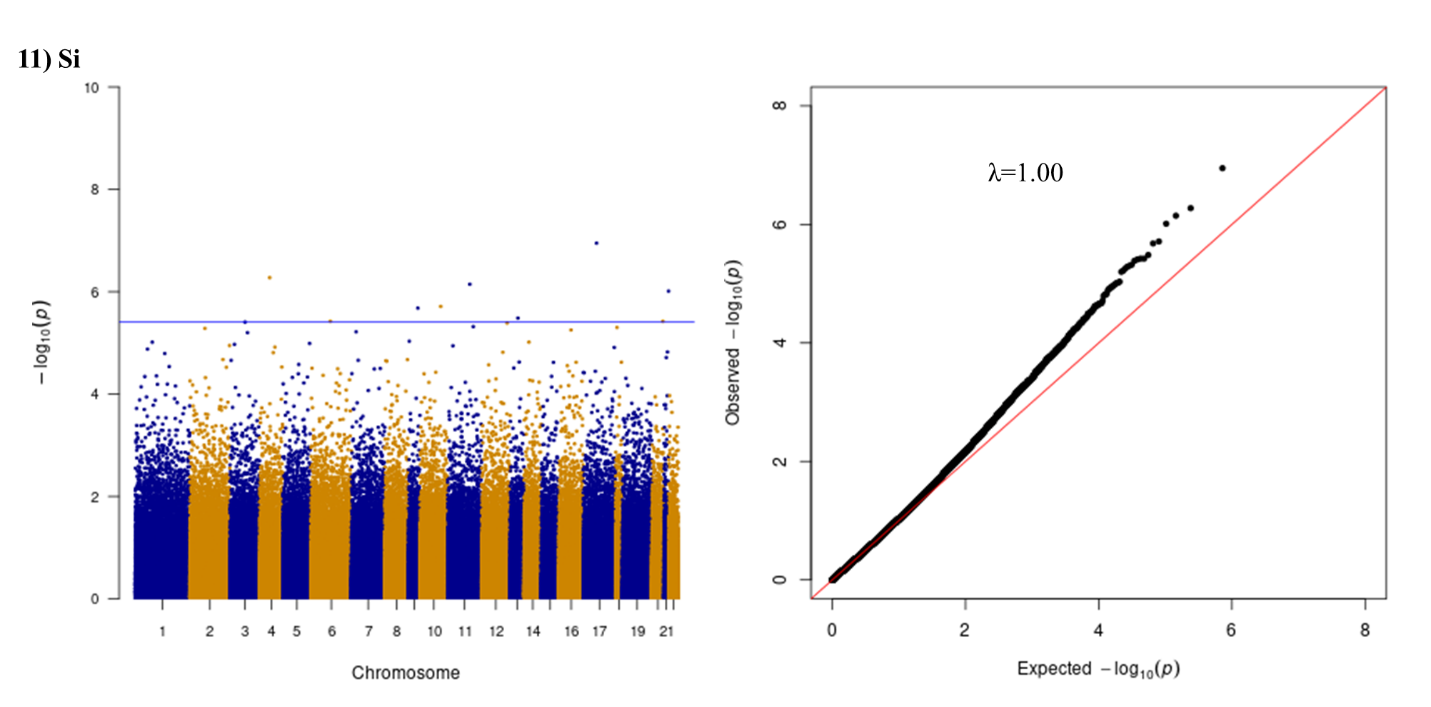


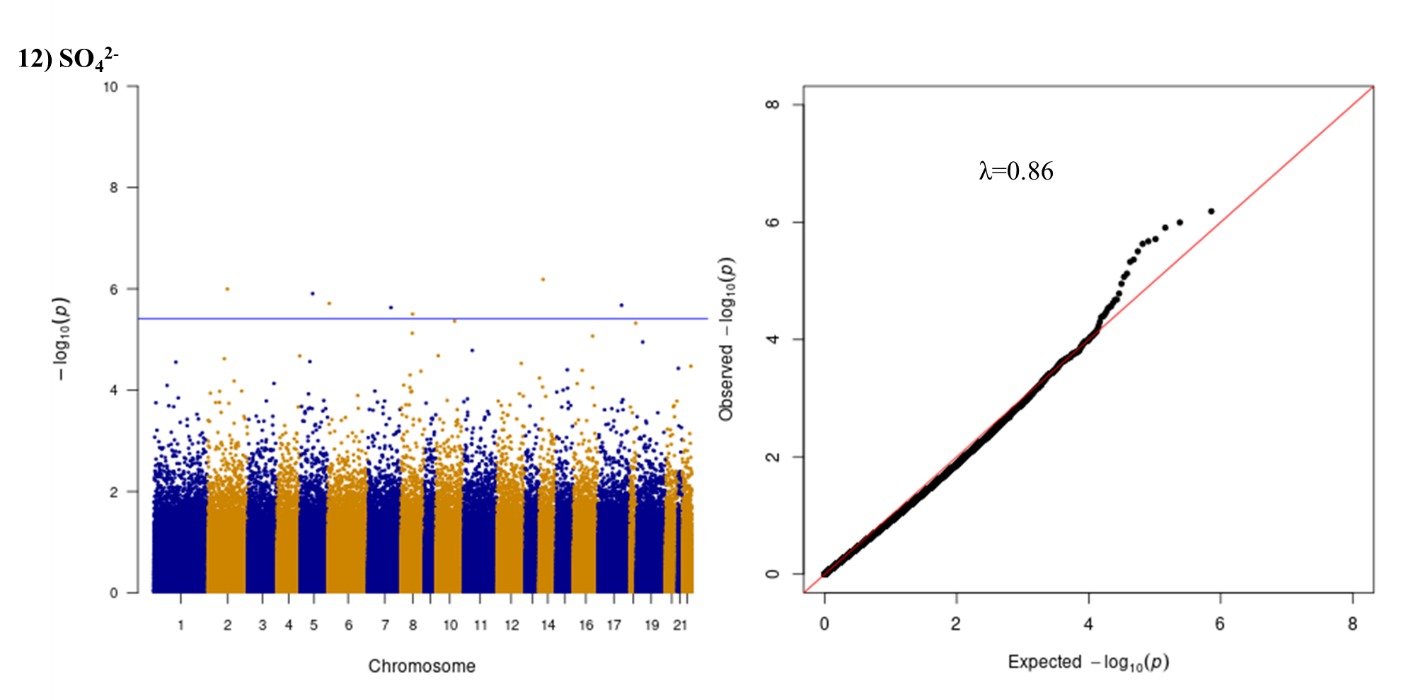


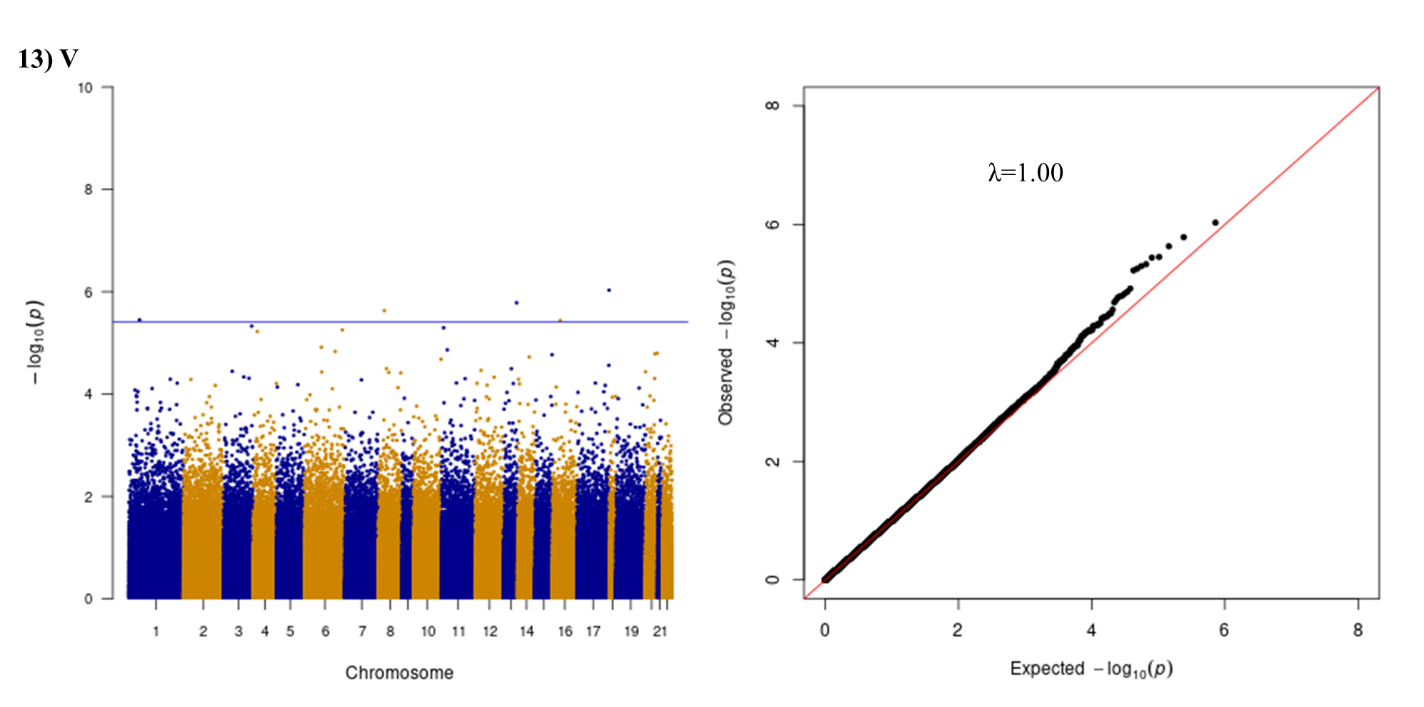


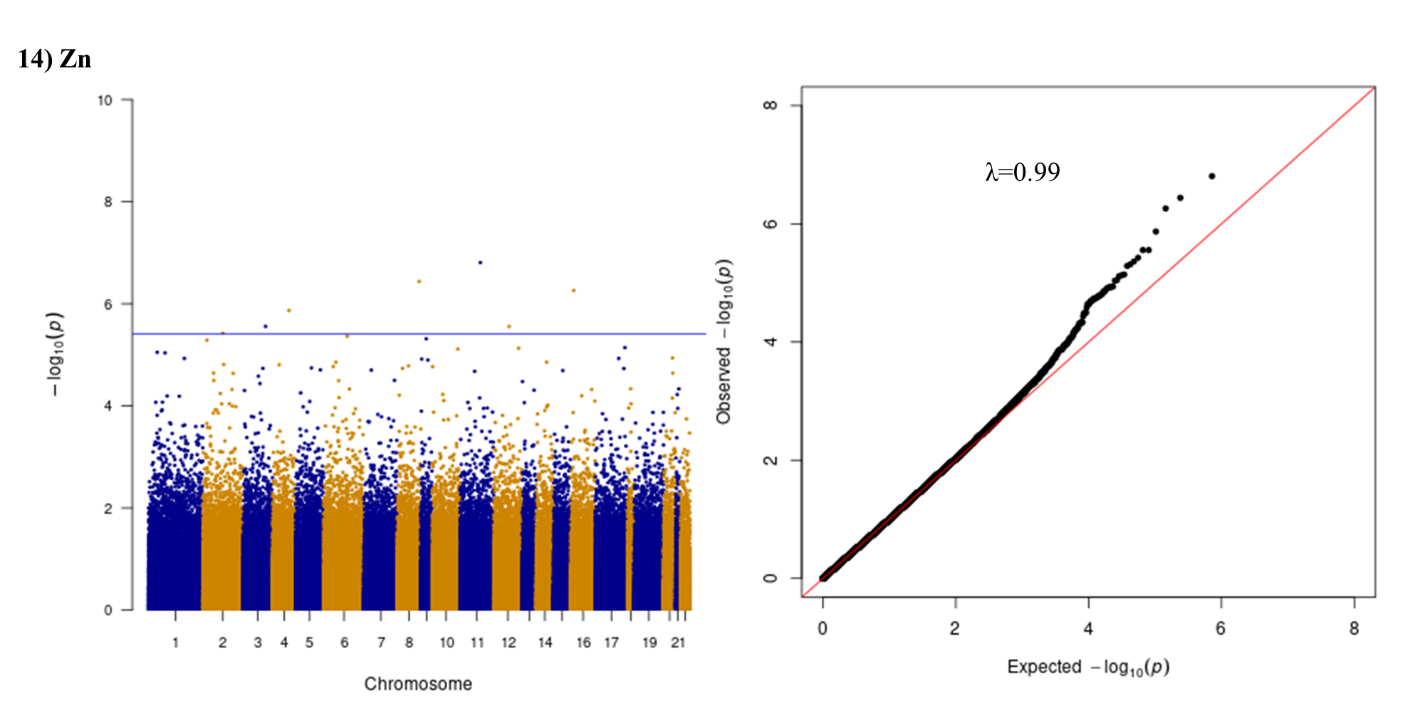


**Figure S3.** Manhattan and quantile-quantile plots with the estimated genomic inflation factor for each cluster in the main analysis.


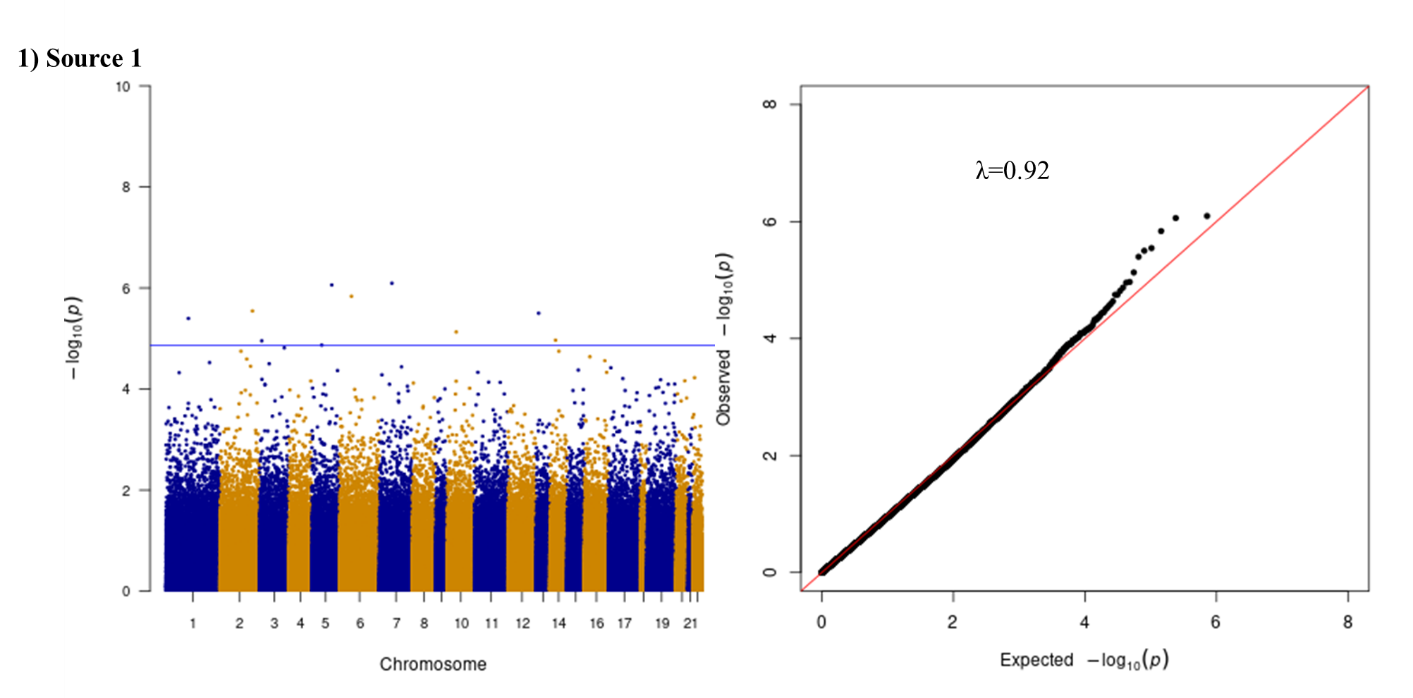


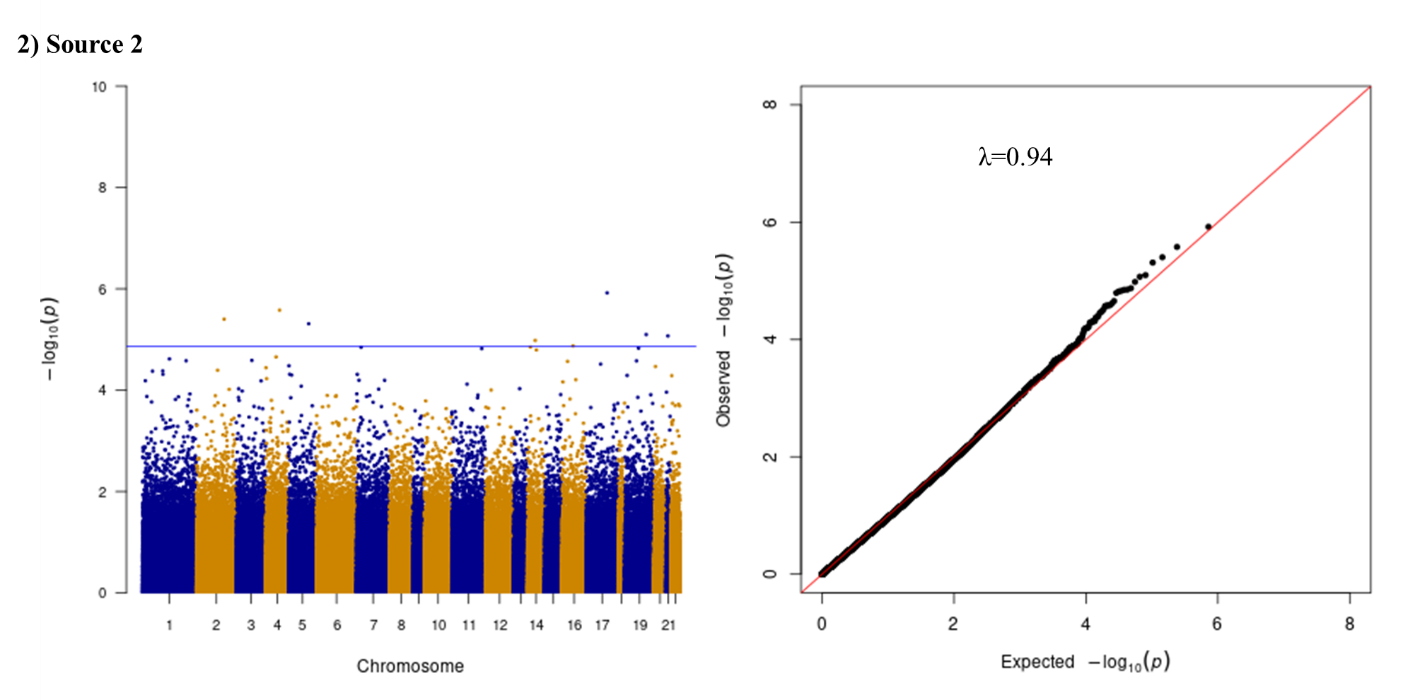


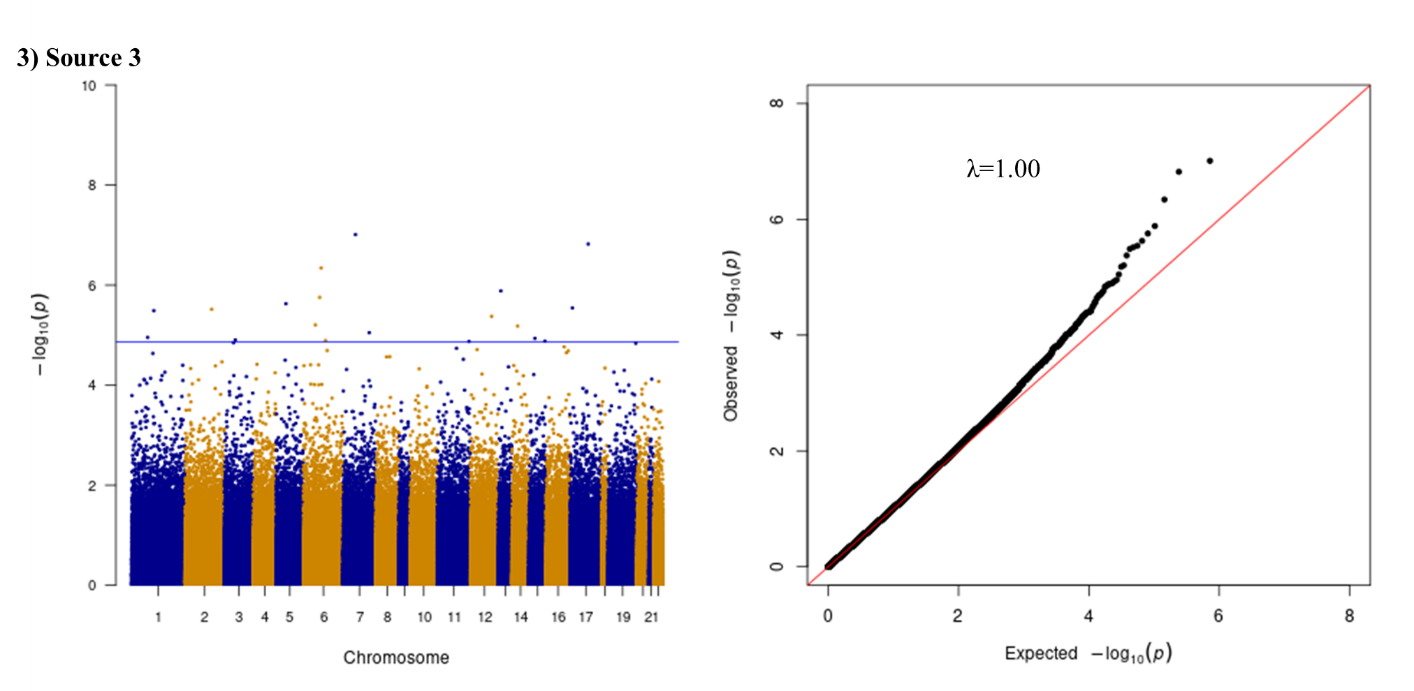


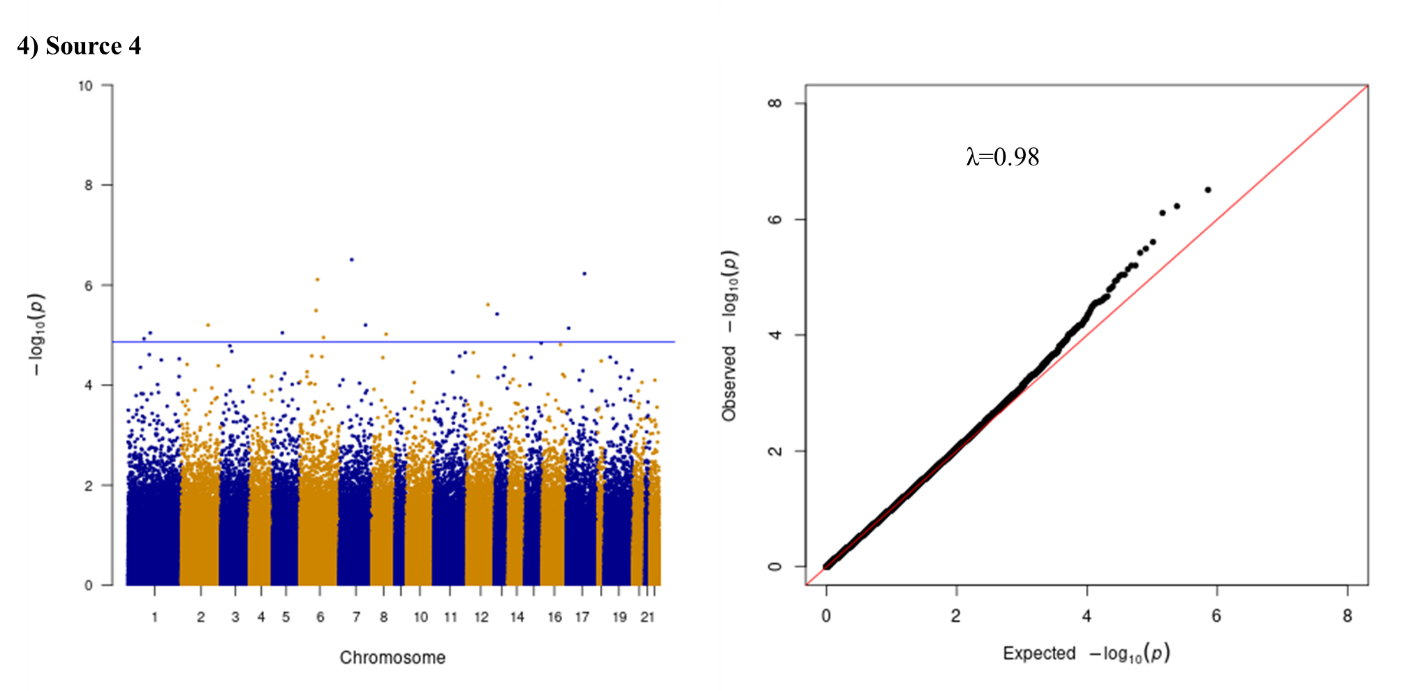


**Figure S4.** Effect size and 95% confidence intervals for the top 5 probes in the main analyses and sensitivity analyses.


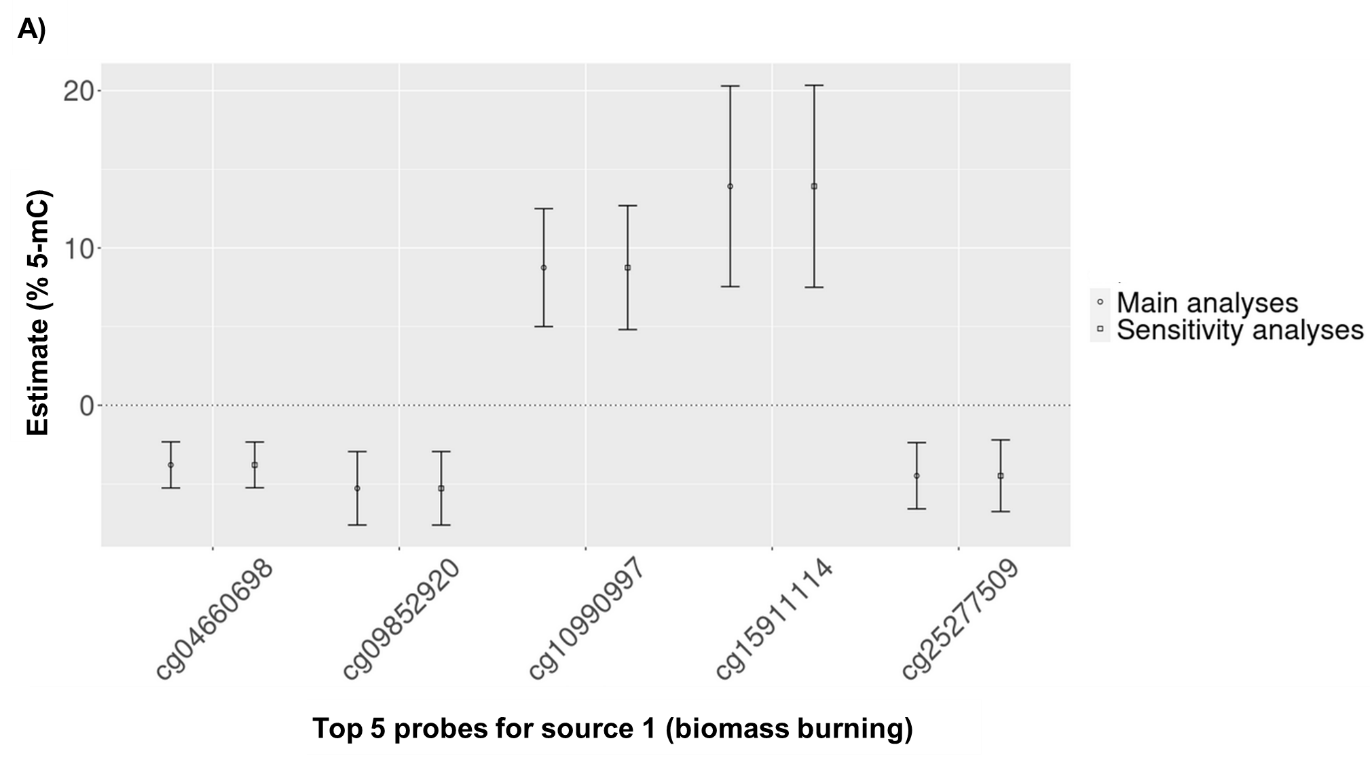


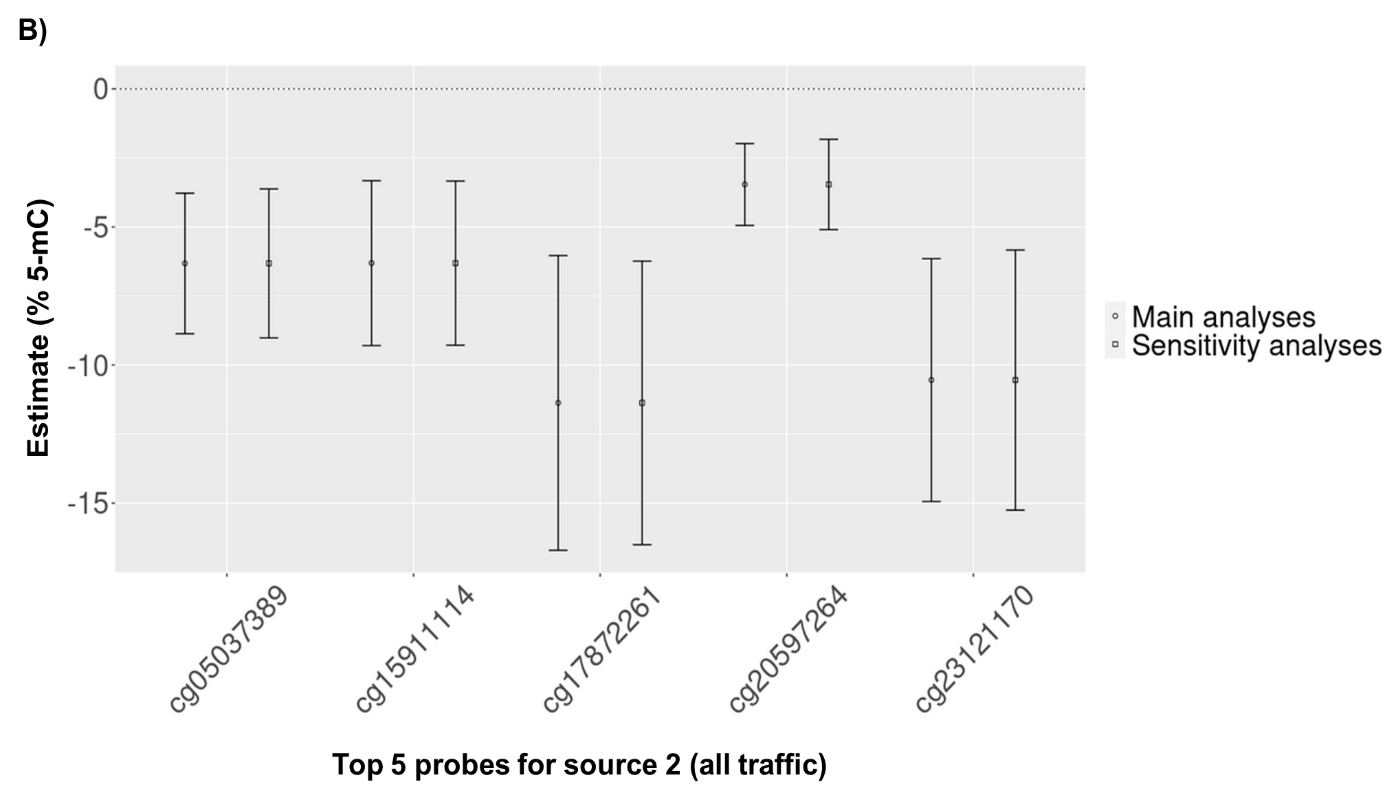


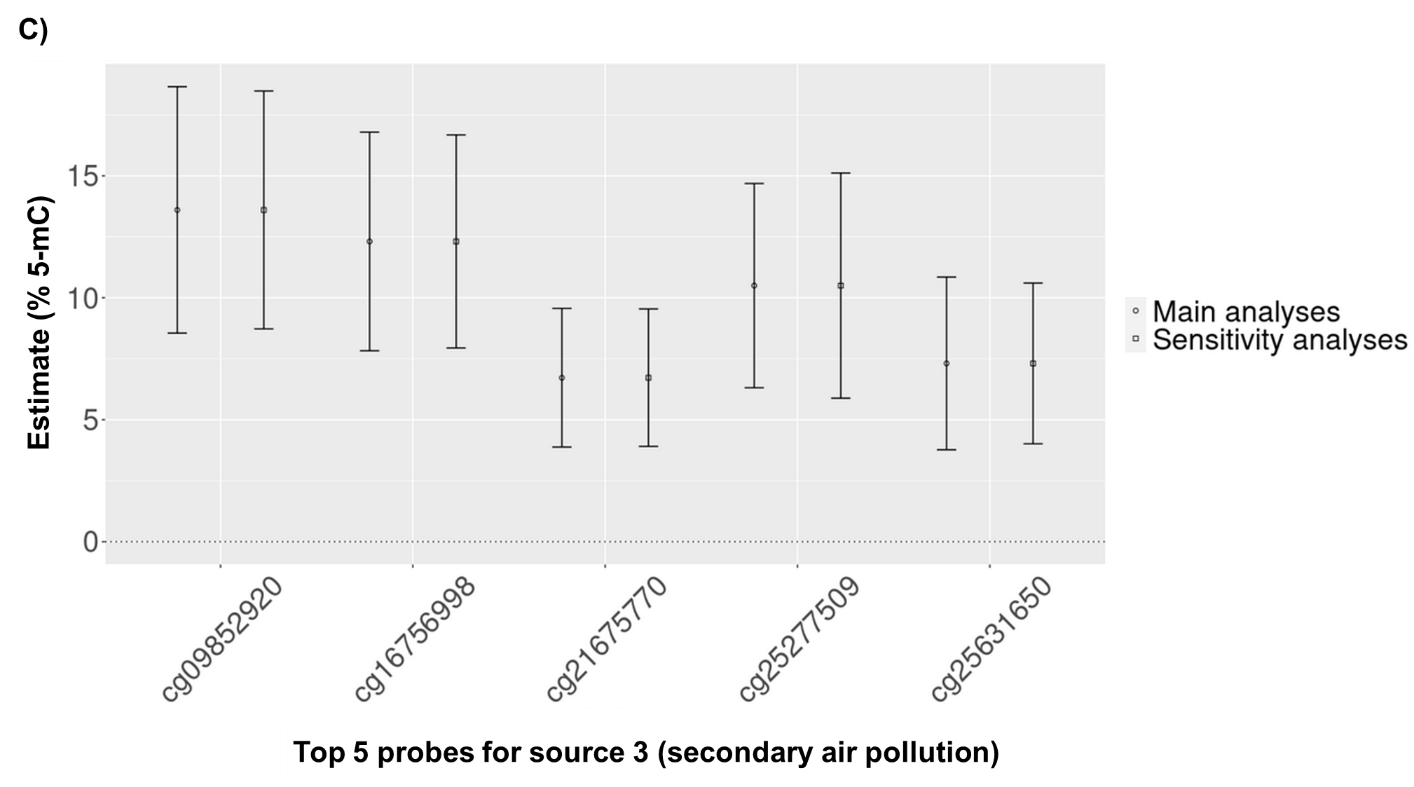


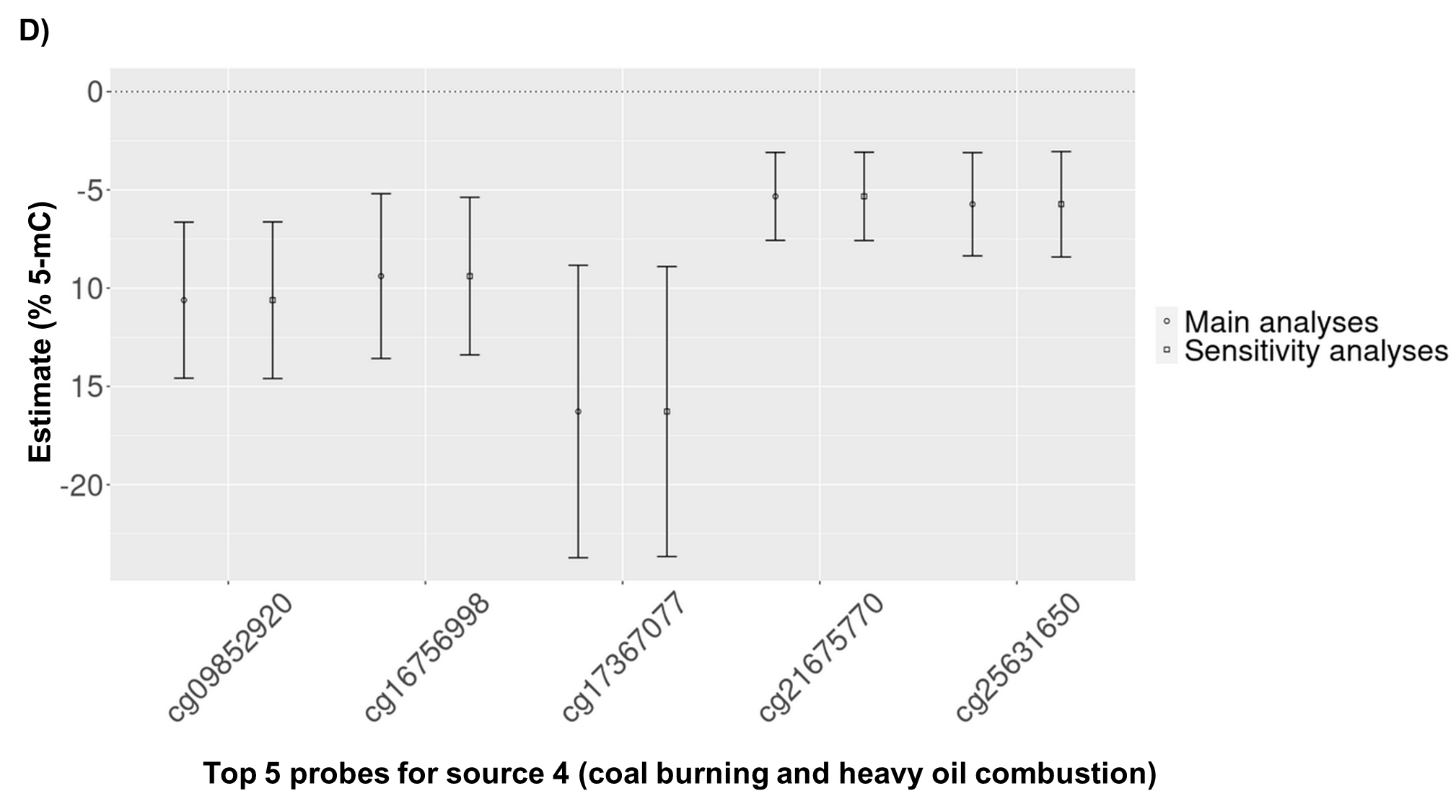

Supplement: Supplementary file 1 — Additional file 1. [file 12940_2023_1007_MOESM1_ESM.zip › Supplementary Information.docx]
